# Tunlametinib (HL-085) plus vemurafenib in patients with advanced *BRAF* V600-mutant solid tumors: an open-label, single-arm, multicenter, phase I study

**DOI:** 10.1186/s40164-024-00528-0

**Published:** 2024-06-12

**Authors:** Yuankai Shi, Xiaohong Han, Qian Zhao, YuLong Zheng, Jianhua Chen, Xinmin Yu, Jian Fang, Yutao Liu, Dingzhi Huang, Tianshu Liu, Hong Shen, Suxia Luo, Hongsheng Yu, Yu Cao, Xi Zhang, Pei Hu

**Affiliations:** 1 Department of Medical Oncology, Beijing Key Laboratory of Clinical Study on Anticancer Molecular Targeted Drugs, National Cancer Center/National Clinical Research Center for Cancer/Cancer Hospital, Chinese Academy of Medical Sciences & Peking Union Medical College, No. 17 Panjiayuan Nanli, Chaoyang District, Beijing, 100021 People’s Republic of China; 2 Clinical Pharmacology Research Center, State Key Laboratory of Complex Severe and Rare Diseases, NMPA Key Laboratory for Clinical Research and Evaluation of Drug, Beijing Key Laboratory of Clinical PK and PD Investigation for Innovative Drugs, Peking Union Medical College Hospital, Chinese Academy of Medical Sciences & Peking Union Medical College, No.1, Shuaifuyuan, Dongcheng District, Beijing, 100730 People’s Republic of China; 3 Department of Oncology, the First Affiliated Hospital of Zhejiang University College of Medicine, Hangzhou, Zhejiang Province 310006 People’s Republic of China; 4 Thoracic Medicine Department I, the Affiliated Cancer Hospital of Xiangya School of Medicine, Hunan Cancer Hospital, Central South University, Changsha, Hunan Province 410006 People’s Republic of China; 5 Department of Oncology, Zhejiang Cancer Hospital, Institute of Basic Medicine and Cancer (IBMC), Chinese Academy of Sciences, Hangzhou, Zhejiang Province 310022 People’s Republic of China; 6 Thoracic Oncology Second Department, Beijing Cancer Hospital, Beijing, 100142 People’s Republic of China; 7 Department of Thoracic Oncology, Key Laboratory of Cancer Prevention and Therapy, Tianjin Medical University Cancer Institute & Hospital, National Clinical Research Center for Cancer, Tianjin’s Clinical Research Center for Cancer, Tianjin, 300060 People’s Republic of China; 8 Department of Medical Oncology, Zhongshan Hospital, Fudan University, Shanghai, 200032 People’s Republic of China; 9 Department of Oncology, the Second Affiliated Hospital of Zhejiang University School of Medicine, Hangzhou, Zhejiang Province 310009 People’s Republic of China; 10 Department of Medical Oncology, Henan Cancer Hospital, the Affiliated Cancer Hospital of Zhengzhou University, Zhengzhou, Henan Province 450008 People’s Republic of China; 11 Department of Radiation Oncology, the Affiliated Hospital of Qingdao University, Qingdao, Shandong Province 266000 People’s Republic of China; 12 Phase I Clinical Research Center, the Affiliated Hospital of Qingdao University, Qingdao, Shandong Province 266000 People’s Republic of China; 13 Department of Clinical Research and Development, Shanghai Kechow Pharma, Inc, Shanghai, 201203 People’s Republic of China

**Keywords:** Tunlametinib, MEK inhibitor, Vemurafenib, *BRAF*, Non-small cell lung cancer, Colorectal cancer

## Abstract

**Background:**

Tunlametinib (HL-085) is a novel, highly selective MEK inhibitor with substantial clinical activities in patients with *NRAS*-mutant melanoma. This phase I study evaluated the safety and preliminary efficacy of tunlametinib plus vemurafenib in patients with advanced *BRAF* V600-mutant solid tumors.

**Methods:**

Patients with confirmed advanced *BRAF* V600-mutant solid tumors who had progressed on or shown intolerance or no available standard therapies were enrolled and received tunlametinib plus vemurafenib. This study consisted of a dose-escalation phase and a dose-expansion phase. Primary end points of this study were safety, the recommended phase II dose (RP2D), and preliminary efficacy.

**Results:**

From August 17, 2018 to April 19, 2022, 72 patients were enrolled. No dose-limiting toxicities occurred, and the maximum tolerated dose was not reached. The RP2D for *BRAF* V600-mutant non-small cell lung cancer (NSCLC) patients was tunlametinib 9 mg plus vemurafenib 720 mg, twice daily (BID, bis in die). Until the data cut-off date of December 15, 2023, of 33 NSCLC patients with evaluable disease, the objective response rate (ORR) was 60.6% (20/33; 95% confidence interval [CI], 42.1–77.1), the median progression free survival (PFS) was 10.5 months (95%CI, 5.6–14.5) and median duration of response (DoR) was 11.3 months (95%CI, 6.8-NE). At the RP2D, ORR was 60.0% (9/15; 95% CI, 32.3–83.7), the median PFS was 10.5 months (95%CI, 5.6 -NE) and median DoR was 11.3 months (95%CI, 3.9-NE). Of 24 colorectal cancer patients with evaluable disease, the ORR was 25.0% (6/24; 95% CI, 5.6-NE). All 72 patients had treatment-related adverse events (TRAEs), and the most common grade 3–4 TRAEs were anemia (*n* = 13, 18.1%) and blood creatine phosphokinase increased (*n* = 10, 13.9%). Tunlametinib was absorbed rapidly with T_max_ of 0.5–1 h. Vemurafeinib did not influence the system exposure of tunlametinib and vice versa, indicating no drug-drug interaction for this combination.

**Conclusions:**

Tunlametinib (HL-085) plus vemurafenib had a favorable safety profile and showed promising antitumor activity in patients with *BRAF* V600-mutant solid tumors. The RP2D for NSCLC was tunlametinib 9 mg BID plus vemurafeinib 720 mg BID.

**Trial Registration:**

ClinicalTrials.gov, NCT03781219.

**Supplementary Information:**

The online version contains supplementary material available at 10.1186/s40164-024-00528-0.

## Background

V-Raf murine sarcoma viral oncogene homolog B (BRAF) is an important protein kinase in the mitogen-activated protein kinase (MAPK) pathway, which plays a critical role in the modulation of cell growth, proliferation, survival, and differentiation; as such, activating *BRAF* mutations are key drivers of oncogenesis [1]. *BRAF* mutations are reported in a variety of human cancers, including melanoma (40-50%) [2], thyroid carcinoma (29-83%) [3], colorectal cancer (CRC; 10-20%) [4], and non-small cell lung cancer (NSCLC; 2-4%) [5]. The most frequent *BRAF* mutation is at *BRAF* V600, which represents a negative prognostic factor in different cancers [3, 6, 7]. For patients with NSCLC or CRC harboring this mutation, chemotherapy or immune checkpoint inhibitor therapy provides limited clinical benefits [7, 8].

Although BRAF inhibitors (BRAFi) as monotherapy showed clinical activity in a fraction of patients with *BRAF* V600-mutant advanced NSCLC, disease progression occurred after a median of 5 to 6.5 months in this patient population [9, 10]. One mechanism underlying acquired resistance and relapse during BRAFi monotherapy is reactivation of the MAPK pathway through a spectrum of genetic alterations or activation of other proteins [11]. The combination of a BRAFi with a MEK inhibitor (MEKi) has shown improved efficacy over BRAF blockade alone in patients with *BRAF* V600-mutant advanced NSCLC, as evidenced by improved tumor response and progression-free survival (PFS) with dabrafenib plus trametinib in this patient population. For pretreated patients, the objective response rate (ORR) and median PFS was 63.9% (95% confidence interval [CI], 46.2–79.2) and 10.2 months (95% CI, 6.9–16.7), respectively. For treatment-naive patients, the ORR and median PFS was 68.4% (95% CI, 54.8–80.1) and 10.8 months (95% CI, 7.0-14.5), respectively [12]. However, the clinical activity of dabrafenib plus trametinib in patients with *BRAF* V600-mutant metastatic CRC was modest (ORR 7%; PFS, 3.5 months [95% CI, 3.4-4.0]) [13], and the current targeted treatment strategy for these patients employs a BRAFi plus an epidermal growth factor receptor (EGFR) inhibitor, which is indicated for use after failure of first-line chemotherapy [14]. It remains to be determined whether alternative BRAFi and MEKi combinations can provide clinical benefits for patients with *BRAF* V600-mutant advanced NSCLC, and other solid tumors.

Tunlametinib (HL-085) is a novel, selective inhibitor of MEK that exhibits high inhibitory activity against MEK1 and moderate activity against MEK2, developed by Shanghai Kechow Pharma, Inc., Shanghai, the People’s Republic of China [15]. In preclinical studies, tunlametinib showed antitumor activity in a variety of tumor cell lines and tumor xenograft models. Tunlametinib blocked proliferation of *RAS/RAF*-mutated cell lines, including A375, Colo 205, Calu-6, and HT29, while showing low antiproliferative activity in normal human cell lines and *RAS/RAF* wild-type H1975 cells. Tunlametinib showed tumor growth inhibition values of 70–76% in a *BRAF*-mutant Colo 205 xenograft model and 60–70% in a *BRAF*-mutant A375 xenograft model [15, 16]. In addition, synergistic antitumor effect was observed when tunlametinib was administered in combination with vemurafenib in an A375 xenograft model (data unpublished). In a previous phase I study of tunlametinib monotherapy in patients with melanoma, a tolerable safety profile was demonstrated with twice daily (bis in die, BID) administration. Tunlametinib concentrations increased in a general dose-proportional manner across the dose range (0.5–18 mg) and showed slight accumulation after multiple dosing [15]. In the same trial, monotherapy of tunlametinib demonstrated good tolerability and clinical benefit, with an ORR of 26.7% and a disease control rate (DCR) of 86.7% in patients with advanced melanoma harboring NRAS mutations [17].

We conducted this phase I study to evaluate the safety, pharmacokinetics (PK), and preliminary efficacy of tunlametinib plus vemurafenib in patients with advanced solid tumors harboring *BRAF* V600 mutations.

## Methods

### Study design and treatment

This open-label, single-arm, multicenter, phase I study was conducted at 10 hospitals in the People’s Republic of China, consisted of a dose-escalation phase and a dose-expansion phase. Here, we report the safety, efficacy and PK results for all patients enrolled in this study.

Dose escalation followed a 3 + 3 design. Based on the PK result of tunlametinib in vivo and in vitro studies, the acute and prolonged toxicity in rodents and non-rodents studies, compared to the efficacy and safety data of congeneric MEK inhibitors, using a quantitative pharmacological calculation method, the starting dose of 0.5 mg BID was established as a safety level in the Phase I trail of its monotherapy in advanced melanoma with *NRAS* mutations [15, 17]. In the tunlametinib monotherapy study, the treatment-related adverse events (TRAEs) leading to permanent discontinuation were interstitial lung disease and retinal artery occlusion in the 18 mg dose group. Therefore, we considered 15 mg as the maximum tolerated dose (MTD) for tunlametinib monotherapy.

In the dose-escalation phase, patients received tunlametinib at dose levels of 0.5, 6, 9, 12, and 15 mg BID, together with vemurafenib 960 mg BID, in 21-day cycles. The Dose-limiting toxicity (DLT) and the MTD were assessed. Detailed definitions are provided in the Supplementary Material (online only).

In the dose-expansion phase, the dose regimens of tunlametinib 12 mg BID plus vemurafenib 960 mg BID, tunlametinib 12 mg BID plus vemurafenib 720 mg BID, andtunlametinib 9 mg BID plus vemurafenib 720 mg BID were further evaluated.

### Patients

Eligible patients were adults with advanced *BRAF* V600-mutant solid tumors in the dose-escalation phase, or *BRAF* V600E-mutant stage IIIB/IIIC/IV NSCLC in the dose-expansion phase. Patients had failed or were intolerant or resistant to standard therapies or had no available standard therapies. Patients were required to have at least one measurable lesion as defined by the Response Evaluation Criteria in Solid Tumors (RECIST) version 1.1, an Eastern Cooperative Oncology Group performance status score of 0 or 1 at study entry, life expectancy of ≥ 3 months, and adequate organ function.

Key exclusion criteria included: prior treatment with specific MEKi or BRAFi; known hypersensitivity to study drug or accessories; active central nervous system metastasis; uncontrolled concomitant diseases or infectious diseases; and use of strong inducers or inhibitors of CYP isozyme within 1 week before study treatment. Details of the inclusion and exclusion criteria are provided in the Supplementary Material (online only).

This study was conducted in compliance with the principles of the Declaration of Helsinki, Good Clinical Practice guidelines, and local applicable regulatory and ethics committee requirements for clinical trials. All patients provided written informed consent before enrollment. The ClinicalTrials.gov registration number is NCT03781219.

### End points

Primary end points were safety, determination of the MTD and DLT in patients with advanced *BRAF* V600-mutant solid tumors, and determination of the recommended phase II dose (RP2D) in patients with advanced *BRAF* V600-mutant NSCLC. Secondary end points included ORR, duration of response (DoR), DCR, PFS, and PK profiles. Definitions of secondary efficacy end points were presented in the Supplementary Material (online only).

### Assessments

Safety assessments included treatment-emergent adverse events (TEAEs), TRAEs, serious adverse events (SAEs), vital signs, physical examinations, and laboratory tests. AEs were graded per the National Cancer Institute Common Terminology Criteria for Adverse Events version 5.0.

Efficacy was evaluated by tumor assessment via computed tomography or magnetic resonance imaging as per the RECIST version 1.1. Tumor assessments were performed at baseline, day 1 of cycle 2 (the time window was ± 3 days), and every 2 cycles thereafter, by the investigators.

PK assessments were performed using a validated ultra-performance liquid chromatography-tandem mass spectrometry method. *BRAF* V600 mutation assessments for patients who had a prior test report were conducted at the study hospitals or qualified independent third-party laboratory as central laboratory, using the histological/cytological methods. Details for measurement of PK is described in the Supplementary Material (online only).

### Sample size estimation

The dose-escalation phase of this study followed the 3 + 3 principle to enroll patients for each dose group, and the sample size depended on occurrence of DLTs and the number of dose groups. Considering *BRAF* as a rare mutation, the China National Medical Products Administration (NMPA) recommended 10–20 patients to be enrolled for the registration trial of safety and efficacy data. Therefore, in the dose-expansion phase of this study, 12 to 24 patients were enrolled for each dose group. Based on the safety, efficacy, and PK results, the expansion cohorts and enrolled patient number were decided by the discussion between the investigator and sponsor.

### Statistical analysis

After all patients have completed at least 12 weeks (4 cycles) of study treatment or discontinued, the primary data analysis will be performed. DLT and MTD were assessed in the DLT analysis set, defined as patients who experienced a DLT during the first cycle or had taken at least 80% of the planned study drug doses and completed all safety evaluations. The safety set (SS) included patients who received at least one dose of study drug. Efficacy analysis set was the full analysis set (FAS), which comprised all patients who received at least one dose of study drug and had baseline data. Efficacy was analyzed in prespecified subgroups: all NSCLC patients, all CRC patients, all papillary thyroid carcinoma (PTC) patients, all melanoma patients and all pancreatic cancer patients in this study. The PK analysis set comprised patients who received at least one dose of study drug, had at least one PK assessment after treatment, and had no major protocol violations that affected the PK evaluation. The incidence and 95% CI of ORR and DCR were estimated using the exact (Clopper-Pearson) method. The median DoR and median PFS were calculated using Kaplan–Meier statistics with 95% CIs. Statistical analyses were conducted using SAS version 9.4 (SAS Institute, Cary, North Carolina, USA).

## Results

### Demographic and baseline characteristics

**Fig. 1:**
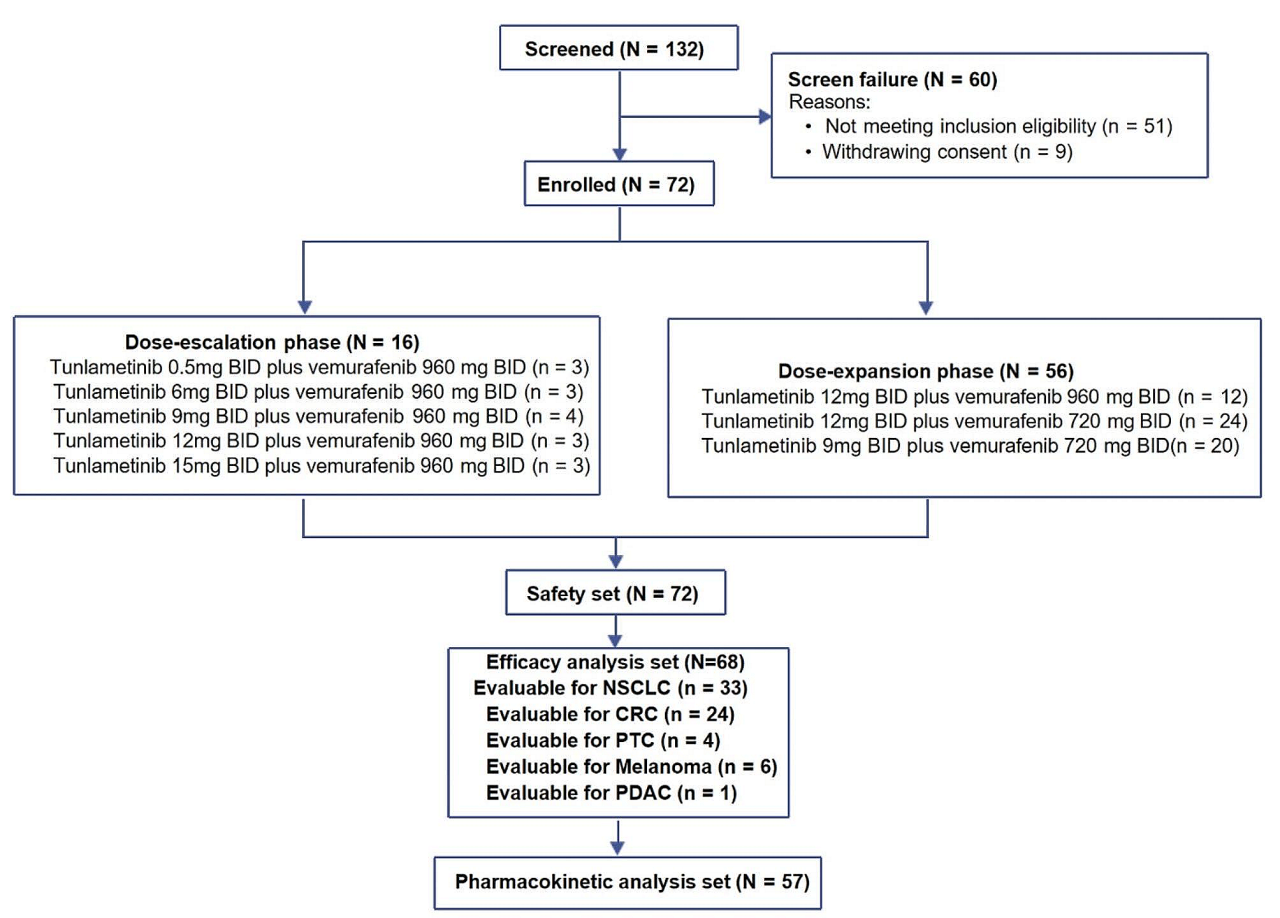
Study design and patient disposition. All dose groups were given study drug treatment twice daily (BID). BID, Bis In Die; CRC, colorectal cancer; NSCLC, non-small cell lung cancer; PTC, papillary thyroid carcinoma; PDAC, pancreatic ductal adenocarcinoma

From August 17, 2018 to April 19, 2022, a total of 72 patients with *BRAF* V600-mutant solid tumors were enrolled; the data cut-off date of this study was December 15, 2023. Patient disposition is presented in Fig. 1, and the demographic and baseline characteristics are presented in Table 1. All patients have completed the 12 weeks of treatment or discontinued treatment caused by disease progression, unacceptable toxicity, withdrawal of consent, or death. The median duration of treatment was 5.3 months (Q1-Q3: 2.7–8.6). Among all patients enrolled, the tumor types included NSCLC (*n* = 36, 50.0%), CRC (*n* = 25, 34.7%), melanoma (*n* = 6, 8.3%), PTC (*n* = 4, 5.6%), and pancreatic cancer (*n* = 1, 1.4%). The median duration of treatment was 4.4 months (Q1-Q3: 2.5–8.2) for tunlametinib and 4.3 months (Q1-Q3: 2.5–7.2) for vemurafenib. Demographic and baseline characteristics by dose group are listed in Supplementary Table S1 (online only).

**Table 1:** Demographic and baseline characteristics

	All Patients	NSCLC Patients	CRC Patients	PTC Patients	Melanoma Patients	PDAC Patients
No. of patients	72	36	25	4	6	1
Age, median (range), years	57 (32–81)	60 (37–81)	53 (32–67)	52(37–68)	53(33–66)	47
Sex, No. (%)						
Male	39 (54.2)	19 (52.8)	15 (60.0)	2 (50.0)	2 (33.3)	1 (100.0)
Female	33 (45.8)	17 (47.2)	10 (40.0)	2 (50.0)	4 (66.7)	0
ECOG PS, No. (%)						
0	32 (44.4)	16 (44.4)	9 (36.0)	3 (75.0)	4 (66.7)	0
1	40 (55.6)	20 (55.6)	16 (64.0)	1 (25.0)	2 (33.3)	1 (100.0)
*BRAF* V600 mutation, No. (%)	72 (100.0)	36 (100.0)	25 (100.0)	4 (100.0)	6 (100.0)	1 (100.0)

Abbreviations: CRC, colorectal cancer; ECOG, Eastern Cooperative Oncology Group; PS, performance status; NSCLC, non-small cell lung cancer; PDAC, pancreatic ductal adenocarcinoma; PTC, papillary thyroid carcinoma

In 36 patients with NSCLC enrolled across the dose-escalation and the dose-expansion phases, the median duration of treatment was 5.7 months (Q1-Q3: 3.7–11.8) for tunlametinib and 5.7 months (Q1-Q3: 3.7–11.0) for vemurafenib. In the FAS of 33 patients with evaluable disease, 45.5% (15/33) patients had received prior systemic antitumor therapy, and 54.5%(18/33) patients were treatment naïve. Demographic and baseline characteristics for the NSCLC cohort by dose group are listed in Supplementary Table S2 (online only).

In 25 patients with CRC, the median duration of treatment was 5.6 months (Q1-Q3: 2.7–7.6) for tunlametinib and 5.6 months (Q1-Q3: 2.7–7.6) for vemurafenib. Twenty-four (96.0%) patients with CRC had received prior antitumor therapy.

Of the 4 patients with PTC, all were radioactive iodine-refractory differentiated thyroid cancer (RAIR-DTC), the median duration of treatment was 4.7 months (Q1-Q3: 1.7–10.3) for tunlametinib and 3.5 months (Q1-Q3: 1.7–6.3) for vemurafenib.

6 patients of Melanoma had all previously treated with systemic therapy (interferon, or dacarbazine plus cisplatin etc.). The median duration of treatment was 2.9 months (Q1-Q3: 0.8-5.0) for tunlametinib and 2.9 months (Q1-Q3: 0.8–7.7) for vemurafenib.

1 patient with pancreatic cancer, whose pathology type was pancreatic ductal adenocarcinoma (PDAC), this patient was previously heavily treated. The median duration of treatment was 3.5 months for both tunlametinib and vemurafenib.

### Safety

No DLTs occurred across all dose groups in the dose-escalation phase, and the MTD was not reached. All 72 patients in the dose-escalation and dose-expansion phases were included in the SS. The most common TRAEs were anemia (61.1%, 44/72), blood creatine phosphokinase increased (56.9%, 41/72), and rash (54.2%, 39/72). The incidence of ≥ grade 3 TRAEs was 59.7% (43/72) and serious TRAEs was 31.9% (23/72; Supplementary Table S3, online only) across all doses. A summary of TRAEs ≥ 15% is listed in Table 2. The most frequent ≥ grade 3 TRAEs were anemia (18.1%, 13/72) and blood creatine phosphokinase increased (13.9%, 10/72). Among all TRAEs, 16.7% (12/72) were reported with ejection fraction decreased, 15.3% (11/72) patients with blurred vision, and 12.5% (9/72) with QT interval prolongation.

**Table 2:** Incidence of TRAEs ≥ 15% (by preferred term)

No. (%)	All Patients (*N* = 72)		NSCLC Patients (*N* = 36)		CRC Patients (*N* = 25)	
	Any Grade	≥ Grade 3	Any Grade	≥ Grade 3	Any Grade	≥ Grade 3
**TRAEs ≥ 15%**						
Anemia	44 (61.1)	13 (18.1)	19 (52.8)	1 (2.8)	19 (76.0)	9 (36.0)
Blood creatine phosphokinase increased	41 (56.9)	10 (13.9)	24 (66.7)	5 (13.9)	10 (40.0)	1 (4.0)
Rash	39 (54.2)	3 (4.2)	18 (50.0)	2 (5.6)	12 (48.0)	2 (8.0%)
Pyrexia	35 (48.6)	2 (2.8)	13 (36.1)	1 (2.8)	17 (68.0)	1 (4.0%)
Aspartate aminotransferase increased	35 (48.6)	2 (2.8)	13 (36.1)	1 (2.8)	13 (52.0)	0 (0.0)
Proteinuria	30 (41.7)	0 (0.0)	12 (33.3)	0 (0.0)	16 (64.0)	0 (0.0)
Peripheral edema	26 (36.1)	1 (1.4)	12 (33.3)	0 (0.0)	8 (32.0)	0 (0.0)
Blood creatinine increased	26 (36.1)	0 (0.0)	11 (30.6)	0 (0.0)	11 (44.0)	0 (0.0)
Facial edema	25 (34.7)	0 (0.0)	15 (41.7)	0 (0.0)	7 (28.0)	0 (0.0)
Alanine aminotransferase increased	23 (31.9)	1 (1.4)	11 (30.6)	1 (2.8)	7 (28.0)	0 (0.0)
Fatigue	23 (31.9)	0 (0.0)	14 (38.9)	0 (0.0)	7 (28.0)	1 (4.0%)
Diarrhea	23 (31.9)	3 (4.2)	8 (22.2)	0 (0.0)	10 (40.0)	2 (8.0%)
Vomiting	20 (27.8)	1 (1.4)	10 (27.8)	1 (2.8)	6 (24.0)	0 (0.0)
Hypoalbuminemia	19 (26.4)	0 (0.0)	8 (22.2)	0 (0.0)	9 (36.0)	0 (0.0)
Blood lactate dehydrogenase increased	17 (23.6)	0 (0.0)	12 (33.3)	0 (0.0)	1 (4.0)	0 (0.0)
Hypertension	15 (20.8)	2 (2.8)	9 (25.0)	1 (2.8)	5 (20.0)	1 (4.0)
Gamma-glutamyltransferase increased	14 (19.4)	2 (2.8)	5 (13.9)	0 (0.0)	5 (20.0)	1 (4.0)
Hypokalemia	14 (19.4)	3 (4.2)	7 (19.4)	0 (0.0)	5 (20.0)	1 (8.0)
Nausea	13 (18.1)	0 (0.0)	7 (19.4)	0 (0.0)	2 (8.0)	0 (0.0)
Mouth ulceration	13 (18.1)	0 (0.0)	9 (25.0)	0 (0.0)	3 (12.0)	0 (0.0)
White blood cell count decreased	12 (16.7)	0 (0.0)	5 (13.9)	0 (0.0)	5 (20.0)	0 (0.0)
Hypocalcemia	12 (16.7)	0 (0.0)	8 (22.2)	0 (0.0)	1 (4.0)	0 (0.0)
Ejection fraction decreased	12 (16.7)	2 (2.8)	3 (8.3)	0 (0.0)	6 (24.0)	1 (4.0)
Dizziness	12 (16.7)	0 (0.0)	8 (22.2)	0 (0.0)	3 (12.0)	0 (0.0)
Protein urine	11 (15.3)	0 (0.0)	6 (16.7)	0 (0.0)	1 (4.0)	0 (0.0)
Blurred vision	11 (15.3)	0 (0.0)	3 (8.3)	0 (0.0)	5 (20.0)	0 (0.0)
Blood bilirubin increased	11 (15.3)	0 (0.0)	6 (16.7)	0 (0.0)	4 (16.0)	0 (0.0)
Blood alkaline phosphatase increased	11 (15.3)	0 (0.0)	5 (13.9)	0 (0.0)	2 (8.0)	0 (0.0)
Neutrophil count decreased	11 (15.3)	2 (2.8)	4 (11.1)	0 (0.0)	4 (16.0)	2 (8.0)
Decreased appetite	10 (13.9)	0 (0.0)	6 (16.7)	0 (0.0)	3 (12.0)	0 (0.0)
Blood glucose increased	10 (13.9)	0 (0.0)	6 (16.7)	0 (0.0)	2 (8.0)	0 (0.0)
Sinus tachycardia	10 (13.9)	0 (0.0)	6 (16.7)	0 (0.0)	2 (8.0)	0 (0.0)
Hyponatremia	9 (12.5)	1 (1.4)	3 (8.3)	0 (0.0)	4 (16.0)	0
Arthralgia	8 (11.1)	0 (0.0)	2 (5.6)	0 (0.0)	4 (16.0)	0
Blood triglycerides increased	8 (11.1)	0 (0.0)	1 (2.8)	0 (0.0)	5 (20.0)	0
Blood myoglobin increased	8 (11.1)	0 (0.0)	7 (19.4)	0 (0.0)	0 (0.0)	0
Platelet count decreased	8 (11.1)	2 (2.8)	1 (2.8)	1 (2.8)	6 (24.0)	1 (4.0)
Eyelid edema	8 (11.1)	0 (0.0)	6 (16.7)	0 (0.0)	1 (4.0)	0
Urinary tract infection	7 (9.7)	1 (1.4)	6 (16.7)	0 (0.0)	1 (4.0)	1 (4.0)
Acneiform dermatitis	7 (9.7)	2 (2.8)	0 (0.0)	0 (0.0)	6 (24.0)	2 (8.0)
Troponin T increased	6 (8.3)	0 (0.0)	6 (16.7)	0 (0.0)	0 (0.0)	0
Headache	6 (8.3)	0 (0.0)	1 (2.8)	0 (0.0)	4 (16.0)	0

NOTE. Incidence of TRAEs ≥ 15% in any group are presented

Abbreviations: CRC, colorectal cancer; NSCLC, non-small cell lung cancer; TEAE, treatment-emergent adverse event; TRAE, treatment-related adverse event

11.1% (8/72) patients experienced TRAEs leading to study drug discontinuation, most occurred in the tunlametinib 12 mg BID plus vemurafenib 960 mg BID and tunlametinib 15 mg BID plus vemurafenib 960 mg BID dose groups (Supplementary Table S3, online only). The most common TRAE leading to study drug discontinuation was ejection fraction decreased (2.8%, 2/72). 6.9% (5/72) patients experienced TEAEs leading to death, including 2 deaths due to poor basic cardiopulmonary function, 2 due to disease progression and 1 due to sudden death. One report of sudden cardiac death was considered related to the study drug treatment of tunlametinib 12 mg BID plus vemurafenib 720 mg BID, as assessed by the investigator. Other deaths were considered not related to the study drug treatment, as assessed by the investigators.

During cycle 2–4, TRAEs leading to study drug discontinuation were reported in 26.7% (4/15) of patients in the tunlametinib 12 mg BID plus vemurafenib 960 mg BID dose group and over 50% of patients experienced a TRAE leading to dose adjustment in the tunlametinib 12 mg BID plus vemurafenib 720 mg BID dose group.

In the tunlametinib 9 mg BID plus vemurafenib 720 mg BID dose group of NSCLC patients, the incidence of ≥ grade 3 TRAEs were 35.3% (6/17) and serious TRAEs were 29.4% (5/17); the ORR, median DoR, and median PFS were 60.0% (95% CI, 32.3–83.7), 11.3 months (95% CI, 3.9-NE), and 10.4 months (95% CI, 5.6-NE), respectively. Therefore, the tunlametinib 9 mg BID plus vemurafenib 720 mg BID group was determined as the RP2D for patients with *BRAF* V600-mutant NSCLC according to the better tolerability and consistent efficacy compared with the other study drug dose groups.

Efficacy.

**Fig. 2:**
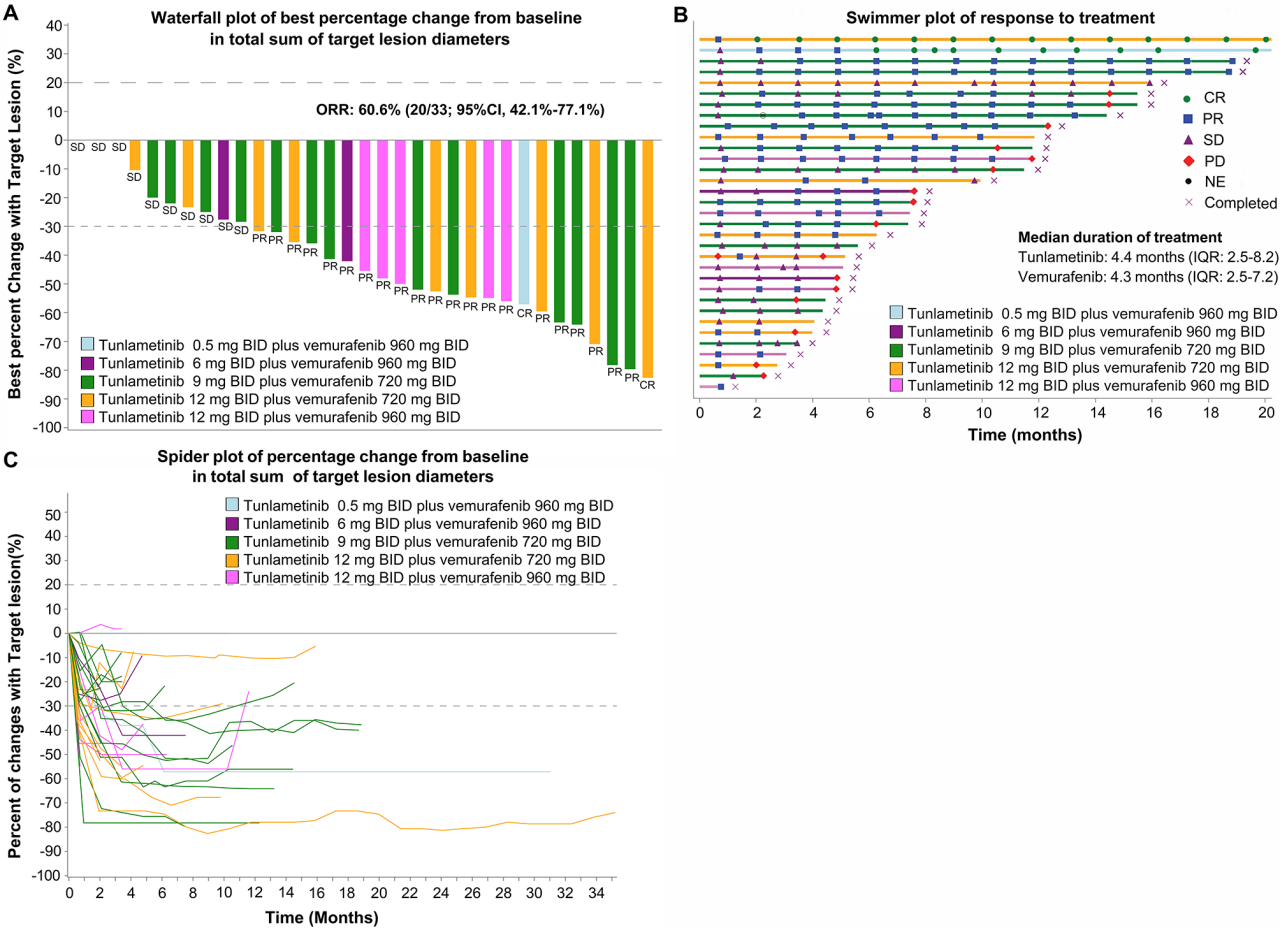
Tumor responses in NSCLC patients with evaluable disease. (**A**) Waterfall plot of best percentage change from baseline in total sum of target lesion diameters. (**B**) Swimmer plot of best percentage change from baseline in total sum of target lesion diameters. (**C**) Spider plot of percentage change from baseline in total sum of target lesion diameters. All dose groups were given study drug treatment twice daily(BID). BID, Bis In Die; CR, complete response; NE, not evaluable; NSCLC, non-small cell lung cancer; PD, progressive disease; PR, partial response; SD, stable disease

**Fig. 3:**
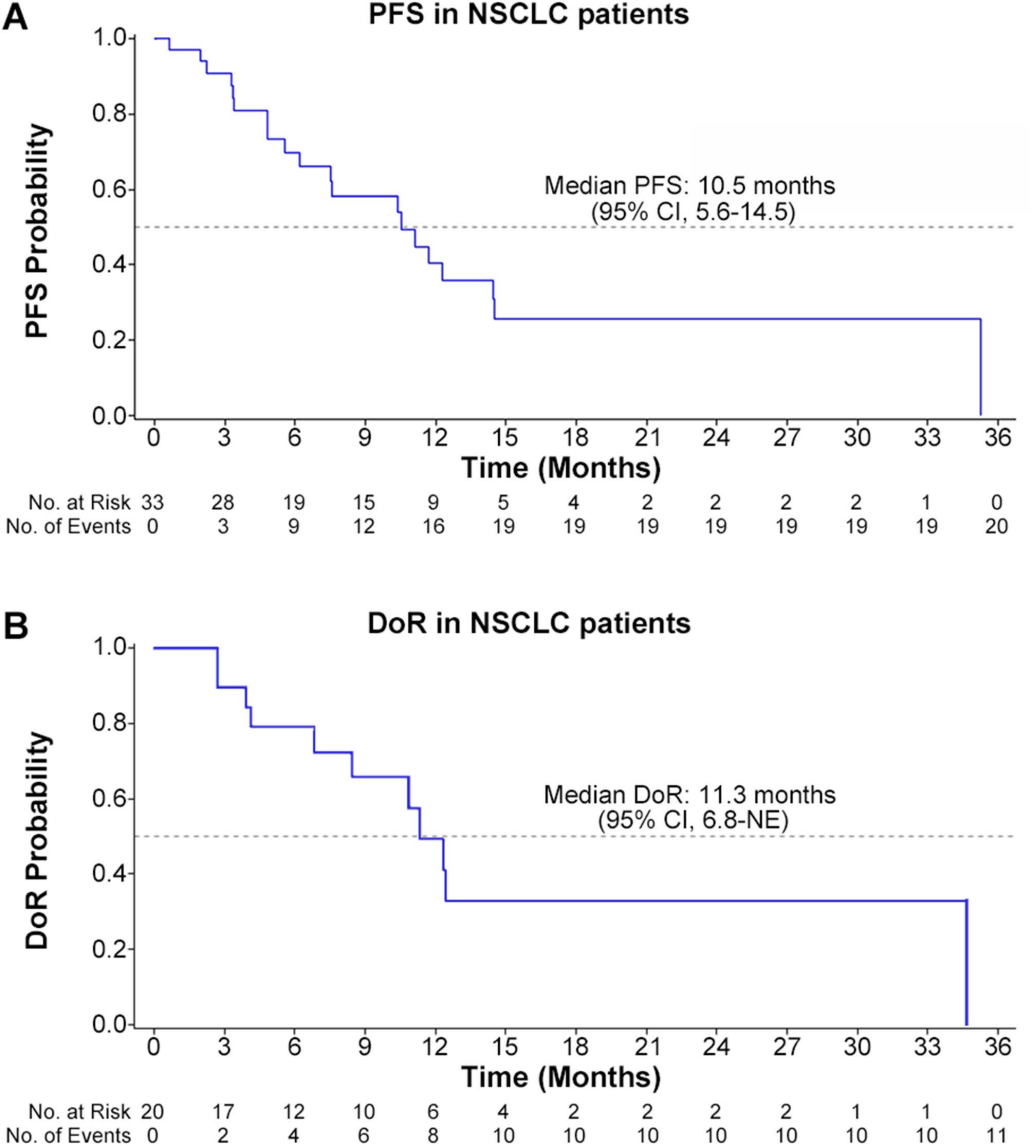
Kaplan–Meier curve for NSCLC patients. (**A**) progression-free survival in NSCLC patients; (**B**) duration of response in NSCLC patients. All dose groups were given study drug treatment twice daily (BID). BID, Bis In Die; CI, confidence interval; DoR, duration of response; NE, not evaluable; NSCLC, non-small cell lung cancer; PFS, progression-free survival

The efficacy outcomes of NSCLC patients are shown in Table 3; Fig. 2, and Fig. 3; efficacy outcomes of CRC, PTC, melanoma and PDAC patients are presented in Supplementary Table S4 and Supplementary Fig S1 and Fig S2 (online only). The swimmer and waterfall plots of all patients are presented in Supplementary Fig S3 (online only).

**Table 3:** Efficacy of tunlametinib plus vemurafenib in NSCLC patients with evaluable disease

Study Drug Dose Groups	0.5 + 960 BID	6 + 960 BID	12 + 960 BID	9 + 720 BID	12 + 720 BID	Total
Tunlametinib dose (mg, BID)	0.5	6	12	9	12	–
Vemurafenib dose (mg, BID)	960	960	960	720	720	–
No. of patients	1	2	6	15	9	33*
ORR, No. (%)	1 (100.0)	1(50.0)	4 (66.7)	9 (60.0)	5 (55.6)	20 (60.6)
CR	1 (100.0)	0	0	0	1 (11.1)	2 (6.1)
PR	0	1 (50.0)	4 (66.7)	9 (60.0)	4 (44.4)	18 (54.5)
SD	0	1 (50.0)	1 (16.7)	5 (33.3)	2 (22.2)	9 (27.3)
PD	0	0	0	1 (6.7)	2 (22.2)	3 (9.1)
NE	0	0	1 (16.7)	0	0	1 (3.0)
DCR, No. (%)	1 (100.0)	2 (100.0)	5 (83.3)	14 (93.3)	7 (77.8)	29 (87.9)
Median DoR, months (95% CI)	–	–	–	11.3 (3.9-NE)	–	11.3 (6.8-NE)
Median PFS, months (95% CI)	–	–	–	10.5 (5.6-NE)	–	10.5 (5.6–14.5)

*: Among 36 NSCLC patients, three patients were excluded from the FAS due to lack of baseline target lesions. 33 patients with baseline target lesions were included in the FAS

Abbreviations: BID, Bis In Die; CI, confidence interval; CR, complete response; DCR, disease control rate; DoR, duration of response; FAS, full analysis set; NE, not evaluable; NSCLC, non-small cell lung cancer; ORR, objective response rate; PD, progressive disease;; PFS, progression-free survival; PR, partial response; SD, stable disease

#### NSCLC patients

Among 36 NSCLC patients, three patients were excluded from the FAS due to lack of baseline target lesions. Of 33 patients with baseline target lesions included in the FAS, 6.1% (2/33) achieved complete response (CR), 54.5% (18/33) achieved partial response (PR), and 27.3% (9/33) experienced stable disease (SD). Patients with CR and PR contributed to an ORR of 60.6% (20/33; 95% CI, 42.1–77.1) and those with CR, PR, and SD contributed to a DCR of 87.9% (29/33; 95% CI, 71.8–96.6) (Table 3). The median DoR was 11.3 months (95% CI, 6.8-not evaluable [NE]); the median PFS was 10.5 months (95% CI, 5.6–14.5) for all NSCLC patients (Fig. 3). For the RP2D of tunlametinib 9 mg BID plus vemurafenib 720 mg BID dose group, the ORR, median DoR, and median PFS were 60.0%, 11.3 months (95% CI, 3.9-NE), and 10.5 months (95% CI, 5.6-NE) respectively (Table 3 and Supplementary Fig S1, online only).

Among 15 patients who had received prior systemic antitumor therapy, 8 achieved PR, the ORR was 53.3% (8/15; 95% CI, 26.6–78.7); in 18 patients who were treatment naïve, 12 achieved PR, the ORR was 66.7% (12/18; 95% CI, 41.0-86.7).

#### CRC patients

Of 24 CRC patients with evaluable disease, six achieved PR and 15 experienced SD. The ORR was 25.0% (6/24; 95% CI, 9.8–46.7) and the DCR was 87.5% (21/24; 95% CI, 67.6–97.3, Supplementary Table S4, online only). The median DoR was 5.5 months (95% CI, 2.9-NE) and the median PFS was 6.2 months (95% CI, 4.8–7.6) (Supplementary Fig S2, online only).

#### PTC patients

Of four patients with PTC, 50.0% (2/4) achieved PR and 50.0% (2/4) experienced SD (Supplementary Table S4 and Fig S1, online only).

In addition, 6 patients with melanoma exhibited varying degrees of tumor shrinkage. And one patient with PDAC achieved PR.

### PK

A total of 57 patients were included in the PK analysis set. After single-dose administration, tunlametinib plasma concentration increased rapidly and declined slowly. The maximum concentration (C_max_) and area under the concentration–time curve (AUC) increased generally in a dose-dependent manner. After multiple administrations, the average accumulation ratio ranged from 0.7 to 3.0 based on C_max_, 1.6 to 4.3 based on AUC_tau_, and 1.6 to 3.5 based on AUC_last_ across doses (Supplementary Table S5, online only).

After single-dose of vemurafenib, vemurafenib was absorbed slowly with median time to maximum plasma concentration (T_max_) of approximately 4 h and then declined slowly. After multiple administrations of vemurafenib, the average accumulation ratio ranged from 8.2 to 16.0 based on C_max_, 13.3 to 14.1 based on AUC_tau_, and 10.6 to 36.3 based on AUC_last_ across doses (Supplementary Table S6, online only).

No drug–drug interaction was identified between tunlametinib and vemurafenib.

## Discussion

This phase I study showed that tunlametinib, a novel, selective inhibitor of MEK, plus vemurafenib was well tolerated and showed preliminary efficacy in patients with advanced *BRAF* V600-mutant solid tumors. No DLTs occurred and the MTD was not reached. Tunlametinib plus vemurafenib showed durable antitumor activity in patients with NSCLC harboring *BRAF* V600 mutations. The RP2D for patients with NSCLC was determined as tunlametinib 9 mg BID plus vemurafenib 720 mg BID according to the current safety and efficacy data. Furthermore, tunlametinib plus vemurafenib demonstrated promising antitumor activity in *BRAF* V600-mutant CRC, PTC, melanoma and pancreatic cancer patients.

The safety profile of tunlametinib plus vemurafenib was consistent with that reported for combinations of the two drug classes. In patients with previously untreated *BRAF* V600-mutant NSCLC, the incidence of grade 3–4 AEs was 69%, and the incidence of AEs leading to dabrafenib plus trametinib treatment discontinuation 22% [18]. In patients with previously treated *BRAF* V600-mutant NSCLC, the incidence of grade 3–4 AEs was 49%, and the incidence of AEs leading to dabrafenib plus trametinib treatment discontinuation was 12% [19]. In patients with NSCLC, the incidence of grade 3–4 AEs was 41%, and the incidence of AEs leading to permanent encorafenib plus binimetinib discontinuation was 15% [20]. Safety concerns with BRAFi plus MEKi combinations include pyrexia, cutaneous, cardiac, and ocular toxicities [21]; these AEs were also observed in this study and were mostly of grade 1 or 2. The most common ≥ grade 3 AEs were hematological abnormalities with tunlametinib plus vemurafenib, which were similar with other BRAFi and MEKi combinations [22] and well managed with dose modifications. Photosensitivity is a common AE associated with vemurafenib, with an incidence of 30% in the phase III trial [23]; incidence was 2.8% and 0% in this study of dose-escalation and dose-expansion phases, respectively. The tunlametinib 9 mg BID plus vemurafenib 720 mg BID dose group (RP2D) of NSCLC patients in this study, the incidence of ≥ grade 3 TRAEs were 35.3% and serious TRAEs were 29.4%, and no treatment discontinuation occurred. This may avoid the occurrence of rapidly acquired resistance caused by vemurafenib monotherapy.

Following the approval of Dabrafenib plus trametinib by the U.S. Food and Drug Administration (FDA) in June 22, 2017, the U.S. FDA approved Encorafenib plus Binimetinib for *BRAF* V600E-mutant NSCLC in October 11, 2023. The results from this study are noteworthy given the new combination treatment options for patients with *BRAF* V600-mutant NSCLC. The efficacy of tunlametinib plus vemurafenib was consistent with that of dabrafenib plus trametinib. For pretreated patients, the ORR and median PFS was 63.9% (95% CI, 46.2–79.2) and 10.2 months (95% CI, 6.9–16.7), respectively. For treatment-naive patients, the ORR and median PFS was 68.4% (95% CI, 54.8–80.1) and 10.8 months (95% CI, 7.0-14.5), respectively [12]. According to the National Comprehensive Cancer Network guideline, single-agent dabrafenib is a treatment option if the combination of dabrafenib and trametinib is not tolerated.Yet dabrafenib monotherapy only showed an ORR of 33% (95% CI, 23–45) and a median PFS of 5.5 (95% CI, 3.4–7.3) months in *BRAF* V600-mutant NSCLC [24]; vemurafenib showed an ORR of 42% (95% CI, 20–67) to 44.9% (95% CI, 35.2–54.8) and a median PFS of 5.2 (95% CI, 3.8–6.8) to 7.3 (95% CI, 3.5–10.8) months in *BRAF*-mutant NSCLC [25, 26]. The efficacy of tunlametinib plus vemurafenib was comparable with that of other BRAFi and MEKi combinations in this patient population. A phase II study of encorafenib plus binimetinib reported an ORR and median PFS of 75% (95% CI, 62–85) NE (95% CI, 15.7-NE) in treatment-naïve patients, respectively, and an ORR and median PFS of 46% (95% CI, 30–63) and 9.3 months (95% CI, 6.2-NE) in previously treated patients, respectively [20]. Tunlametinib plus vemurafenib demonstrated favorable antitumor activity and has the potential to be a new combination regimen for the treatment of *BRAF* V600-mutant NSCLC.

The efficacy of BRAFi and MEKi combinations in patients with *BRAF* V600-mutant CRC has not yet been established, and chemotherapy remains the mainstay first-line treatment for patients with *BRAF* V600-mutant CRC [14]. An emerging targeted treatment strategy for *BRAF* V600-mutant CRC relies on additional blockade of EGFR; however, this is used in the second-line setting only [14]. This study demonstrated encouraging antitumor activity with tunlametinib plus vemurafenib in patients with *BRAF* V600-mutant CRC, with an ORR of 25.0% (95% CI, 9.8–46.7) and a DCR of 87.5% (95% CI, 67.6–97.3). These results indicate that targeting BRAF and MEK is also a viable strategy in patients with *BRAF* V600-mutant CRC. Furthermore, dabrafenib monotherapy showed an ORR of 35% (95% CI, 17–56) in patients with *BRAF* V600-mutant PTC [27]; the antitumor activity of tunlametinib plus vemurafenib in patients with *BRAF* V600-mutant PTC is effective, 2 patients achieved PR among 4 patients.

The PK profiles of tunlametinib and vemurafenib were consistent with previous studies [15, 28]. PK parameters of tunlametinib and vemurafenib were comparable when administered in combination, compared with when administered alone. No drug–drug interaction was observed, possibly due to the different metabolic pathways of tunlametinib and vemurafenib. Tunlametinib is mainly metabolized by CYP2C9, while vemurafenib is metabolized mainly by CYP3A4 [29], and neither are inducers nor inhibitors for each other.

In this study, tunlametinib plus vemurafenib showed favorable safety and antitumor activity in patients with *BRAF* V600-mutant NSCLC, CRC, and PTC. However, we are aware of several limitations, the results of which should be interpreted with caution given the small sample size in each cohort. The RP2D was not determined for patients with *BRAF* V600-mutant CRC and PTC. Larger prospective studies of patients with *BRAF* V600-mutant NSCLC, CRC, and PTC are needed to confirm the efficacy of this combination treatment.

In summary, tunlametinib plus vemurafenib had an acceptable risk-benefit profile, and all AEs were manageable. This study showed promising antitumor activity of tunlametinib plus vemurafenib in the treatment of patients with *BRAF* V600-mutant NSCLC, CRC and PTC. Hence, we undertake the phase II study of tunlametinib 9 mg BID plus vemurafenib 720 mg BID in patients with *BRAF* V600-mutant NSCLC (NCT05900219), and we will further investigate the efficacy and safety of tunlametinib plus vemurafenib in patients with *BRAF* V600-mutant CRC, PTC and other solid tumors.

Over the past 20 years, small-molecule tyrosine kinase inhibitors have changed the treatment landscape of advanced solid tumors [30, 31]. As a target of the MAPK pathway, MEK inhibitors have great potential. It can be combined not only with BRAF inhibitors, but also with PD-1 [32], chemotherapy, etc [33]. Dabrafenib plus trametinib have been administrated for melanoma, NSCLC, thyroid cancer, but also for endometrial cancer [34], etc. Meanwhile, MEK inhibitors monotherapy can also be used for BRAF non-V600 mutations, such as K601E mutations [35]. We believe that tunlametinib has great potential for development and broad prospects, the study of tunlametinib plus vemurafenib for pan-tumor patients with BRAF V600E-mutant, as well as the study of tunlametinib combined with immune checkpoint inhibitors, chemotherapy and more for further research.

### Electronic supplementary material

Below is the link to the electronic supplementary material.

Supplementary Material 1

## Data Availability

No datasets were generated or analysed during the current study.

## Declarations

### Prior presentation

Previously, part of the results of this study have been presented as a poster (poster No.3973) at the 2023 European Society for Medical Oncology Congress, Madrid, Spain, October 20–24, 2023.

## References

[1] Wellbrock C, Karasarides M, Marais R (2004). The RAF proteins take centre stage. Nat Rev Mol Cell Biol.

[2] Jakob JA, Bassett RL, Ng CS, Curry JL, Joseph RW, Alvarado GC, Rohlfs ML, Richard J, Gershenwald JE, Kim KB (2012). NRAS mutation status is an independent prognostic factor in metastatic melanoma. Cancer.

[3] Lassalle S, Hofman V, Ilie M, Butori C, Bozec A, Santini J, Vielh P, Hofman P (2010). Clinical impact of the detection of BRAF mutations in thyroid pathology: potential usefulness as diagnostic, prognostic and theragnostic applications. Curr Med Chem.

[4] Lochhead P, Kuchiba A, Imamura Y, Liao X, Yamauchi M, Nishihara R, Qian ZR, Morikawa T, Shen J, Meyerhardt JA (2013). Microsatellite instability and BRAF mutation testing in colorectal cancer prognostication. J Natl Cancer Inst.

[5] Paik PK, Arcila ME, Fara M, Sima CS, Miller VA, Kris MG, Ladanyi M, Riely GJ (2011). Clinical characteristics of patients with lung adenocarcinomas harboring BRAF mutations. J Clin Oncol.

[6] Marchetti A, Felicioni L, Malatesta S, Grazia Sciarrotta M, Guetti L, Chella A, Viola P, Pullara C, Mucilli F, Buttitta F (2011). Clinical features and outcome of patients with non-small-cell lung cancer harboring BRAF mutations. J Clin Oncol.

[7] De Roock W, Claes B, Bernasconi D (2010). Effects of KRAS, BRAF, NRAS, and PIK3CA mutations on the efficacy of cetuximab plus chemotherapy in chemotherapy-refractory metastatic colorectal cancer: a retrospective consortium analysis. Lancet Oncol.

[8] Barlesi F, Mazieres J, Merlio JP,DebieuvreD,Mosser J, LenaH (2016). Routine molecular profiling of patients with Advanced Non-small-cell Lung Cancer: results of a 1-Year Nationwide Programme of the French Cooperative Thoracic Intergroup (IFCT). Lancet.

[9] Mazieres J, Cropet C, Montane L, Barlesi F, Souquet PJ, Quantin X, Dubos-Arvis C, Otto J, Favier L, Avrillon V (2020). Vemurafenib in non-small-cell lung cancer patients with BRAF(V600) and BRAF(nonV600) mutations. Ann Oncol.

[10] Subbiah V, Gervais R, Riely G, Hollebecque A, Blay JY, Felip E, Schuler M, Goncalves A, Italiano A, Keedy V et al. Efficacy of vemurafenib in patients with non-small-cell lung cancer with BRAF V600 mutation: An open-label, single-arm cohort of the histology-independent VE-BASKET study. JCO Precis Oncol. 2019;3:PO.18.00266. 10.1200/PO.18.00266 PMC7446432 32914022

[11] Shi H, Hugo W, Kong X, Hong A, Koya RC, Moriceau G, Chodon T, Guo R, Johnson DB, Dahlman KB (2014). Acquired resistance and clonal evolution in melanoma during BRAF inhibitor therapy. Cancer Discov.

[12] Planchard D, Besse B, Groen HJM, Hashemi SMS, Mazieres J, Kim TM, Quoix E, Souquet PJ, Barlesi F, Baik C (2022). Phase 2 study of dabrafenib plus trametinib in patients with BRAF V600E-mutant metastatic NSCLC: updated 5-year survival rates and genomic analysis. J Thorac Oncol.

[13] Corcoran RB, Atreya CE, Falchook GS, Kwak EL, Ryan DP, Bendell JC, Hamid O, Messersmith WA, Daud A, Kurzrock R (2015). Combined BRAF and MEK inhibition with dabrafenib and trametinib in BRAF V600-mutant colorectal cancer. J Clin Oncol.

[14] National Comprehensive Cancer Network. NCCN guidelines: Colon cancer. Version 3.2022. https://www.nccn.org/guidelines/guidelines-detail?category=1&id=1428. 2022.

[15] Zhao Q, Wang T, Wang H, Cui C, Zhong W, Fu D, Xi W, Si L, Guo J, Cheng Y (2022). Phase I pharmacokinetic study of an oral, small-molecule MEK inhibitor tunlametinib in patients with advanced NRAS mutant melanoma. Front Pharmacol.

[16] Cheng Y, Tian H (2017). Current development status of MEK inhibitors. Molecules.

[17] Wang X, Luo Z, Chen J, Chen Y, Ji D, Fan L, Chen L, Zhao Q, Hu P, Sun P (2023). First-in-human phase I dose-escalation and dose-expansion trial of the selective MEK inhibitor HL-085 in patients with advanced melanoma harboring NRAS mutations. BMC Med.

[18] Planchard D, Smit EF, Groen HJM, Mazieres J, Besse B, Helland A, Giannone V, D’Amelio AM, Zhang P, Mookerjee B (2017). Dabrafenib plus Trametinib in patients with previously untreated BRAF(V600E)-mutant metastatic non-small-cell lung cancer: an open-label, phase 2 trial. Lancet Oncol.

[19] Planchard D, Besse B, Groen HJM, Souquet PJ, Quoix E, Baik CS, Barlesi F, Kim TM, Mazieres J, Novello S (2016). Dabrafenib plus Trametinib in patients with previously treated BRAF(V600E)-mutant metastatic non-small cell lung cancer: an open-label, multicentre phase 2 trial. Lancet Oncol.

[20] Riely GJ, Smit EF, Ahn MJ, Felip E, Ramalingam SS, Tsao A, Johnson M, Gelsomino F, Esper R, Nadal E et al. Phase II, open-label study of encorafenib plus binimetinib in patients with BRAF(V600)-mutant metastatic non-small-cell lung cancer. J Clin Oncol. 2023:JCO2300774. 10.1200/JCO.23.00774 37270692

[21] Heinzerling L, Eigentler TK, Fluck M, Hassel JC, Heller-Schenck D, Leipe J, Pauschinger M, Vogel A, Zimmer L, Gutzmer R (2019). Tolerability of BRAF/MEK inhibitor combinations: adverse event evaluation and management. ESMO open.

[22] Garutti M, Bergnach M, Polesel J, Palmero L, Pizzichetta MA, Puglisi F (2022). BRAF and MEK inhibitors and their toxicities: a meta-analysis. Cancers (Basel).

[23] Chapman PB, Hauschild A, Robert C, Haanen JB, Ascierto P, Larkin J, Dummer R, Garbe C, Testori A, Maio M (2011). Improved survival with vemurafenib in melanoma with BRAF V600E mutation. N Engl J Med.

[24] Planchard D, Kim TM, Mazieres J, Quoix E, Riely G, Barlesi F, Souquet PJ, Smit EF, Groen HJ, Kelly RJ (2016). Dabrafenib in patients with BRAF(V600E)-positive advanced non-small-cell lung cancer: a single-arm, multicentre, open-label, phase 2 trial. Lancet Oncol.

[25] Mazieres J, Cropet C, Montané L (2020). Vemurafenib in non-small-cell lung cancer patients with BRAFV600 and BRAFnonV600 mutations. Ann Oncol.

[26] Hyman DM, Puzanov I, Subbiah V et al. Vemurafenib in Multiple Nonmelanoma Cancers with BRAF V600 Mutations [published correction appears in N Engl J Med. 2018;379(16):1585]. N Engl J Med. 2015;373(8):726–736. 10.1056/NEJMoa1502309 PMC4971773 26287849

[27] Busaidy NL, Konda B, Wei L, Wirth LJ, Devine C, Daniels GA, DeSouza JA, Poi M, Seligson ND, Shah MH et al. Dabrafenib versus Dabrafenib + Trametinib in -mutated Radioactive Iodine Refractory differentiated thyroid Cancer: results of a Randomized, phase 2. Open-Label Multicenter Trial Thyroid 2022;32(10). 10.1089/thy.2022.0115 PMC9595631 35658604

[28] Si L, Zhang X, Xu Z, Jiang Q, Bu L, Wang X, Mao L, Zhang W, Richie N, Guo J (2018). Vemurafenib in Chinese patients with BRAF(V600) mutation-positive unresectable or metastatic melanoma: an open-label, multicenter phase I study. BMC Cancer.

[29] United States Food and Drug Administration. ZELBORAF® (vemurafenib) tablet for oral use. https://www.accessdata.fda.gov/drugsatfda_docs/label/2017/202429s012lbl.pdf.

[30] Wu Q, Qian W, Sun X, Jiang S (2022). Small-molecule inhibitors, immune checkpoint inhibitors, and more: FDA-approved novel therapeutic drugs for solid tumors from 1991 to 2021. J Hematol Oncol.

[31] Huang L, Jiang S, Shi Y (2020). Tyrosine kinase inhibitors for solid tumors in the past 20 years (2001–2020). J Hematol Oncol.

[32] Shuyu D, Li A, Martial AB, Schrock, Jane J (2017). Liu. Extraordinary clinical benefit to sequential treatment with targeted therapy and immunotherapy of a BRAF V600E and PD-L1 positive metastatic lung adenocarcinoma. Experimental Hematol Oncol.

[33] Jing Han Y, Liu S, Yang X, Wu HL (2021). Qiming Wang. MEK inhibitors for the treatment of non-small cell lung cancer. J Hematol Oncol.

[34] Moschetta M, Mak G, Hauser J, Davies C (2017). Mario Uccello and Hendrik-Tobias Arkenau. Dabrafenib and trametinib activity in a patient with BRAF V600E mutated and microsatellite instability high (MSI-H) metastatic endometrial cancer. Experimental Hematol Oncol.

[35] Riccardo Marconcini L, Galli A, Antonuzzo S, Bursi C, Roncella G, Fontanini (2017). Elisa Sensi and Alfredo Falcone. Metastatic BRAF K601E-mutated melanoma reaches complete response to MEK inhibitor trametinib administered for over 36 months. Experimental Hematol Oncol.

