# Supplementary material for: Tunlametinib (HL-085) plus vemurafenib in patients with advanced BRAF V600-mutant solid tumors: an open-label, single-arm, multicenter, phase I study
Source: Exp Hematol Oncol. 2024 Jun 12;13:60. doi: 10.1186/s40164-024-00528-0 (PMC11167782; doi:10.1186/s40164-024-00528-0)

# SUPPLEMENTARY MATERIALS

Table of contents

Definitions of MTD and DLT

Inclusion and exclusion criteria

Definition of secondary efficacy end points

Measurement of PK

Supplementary Table S1. Demographic and Baseline Characteristics of All Patients with Solid Tumors by Dose Group

Supplementary Table S2. Demographic and Baseline Characteristics of NSCLC Patients by Dose Group

Supplementary Table S3. AE Summary of All, NSCLC, and CRC Patients

Supplementary Table S4. Antitumor activities of Tunlametinib/Vemurafenib in CRC and PTC Patients with Evaluable Disease

Supplementary Table S5. PK parameters of Tunlametinib (0.5mg-15mg)

Supplementary Table S6. PK parameters of Vemurafenib (720mg-960mg)

Supplementary Figure S1. Tumor responses in patient with evaluable disease, non-small-cell lung cancer, colorectal cancer, and papillary thyroid carcinoma. Swimmer plot of treatment duration of (A) tunlametinib 9 mg plus vemurafenib 720 mg group in NSCLC patients, (B) CRC patients, and (C) PTC patients. Waterfall plot of best percent change from baseline in total sum of target lesion diameters of (D) tunlametinib 9 mg plus vemurafenib 720 mg group in NSCLC patients, (E) CRC patients, and (F) PTC patients.

Supplementary Figure S2. Kaplan-Meier Plot of (A) progression-free survival and (B) duration of response in CRC patients.

Supplementary Figure S3. Responses of all patients with evaluable disease in all patients with solid tumors. (A) Swimmer plot of treatment duration. (B) Waterfall plot of best percent change from baseline in total sum of target lesion diameters. CR, complete response; PD, progressive disease; PR, partial response; SD, stable disease.

## Definitions of MTD and DLT

Maximum tolerated dose (MTD) was defined as the highest dose at which the DLT rate was < 33%. Dose-limiting toxicity (DLT) was defined as study treatment related toxicity during the first cycle, including grade ≥ 4 hematological toxicity, grade ≥3 hemorrhagic thrombocytopenia, or grade ≥3 febrile neutropenia; grade ≥ 3 non-hematological toxicity that did not recover to grade ≤ 2 or dose interruption due to toxicity ≥ 14 days, such as nausea, vomiting, skin rash, diarrhea, abnormalities of liver enzymes and total bilirubin, etc.; grade ≥ 3 eye disorder that did not recover to grade ≤ 2 or dose interruption due to toxicity ≥ 14 days; grade ≥ 3 abnormal cardiac function that did not recover to grade ≤ 2 or dose interruption due to toxicity ≥ 14 days, such as abnormal blood pressure, electrocardiogram, and left ventricular ejection fraction.

## Inclusion and exclusion criteria

Inclusion criteria

1. Adult men or women (18 years of age or older).
2. Patients with advanced solid tumor had failed, intolerant, or resisted to standard therapies, or had no available standard therapies (NSCLC with stage IIIb-IV for phase 1b study).
3. Had confirmed advanced BRAF V600-mutated solid tumor with prior gene test report or provided adequate sample for histological/cytological tests at baseline (patients BRAF V600E-mutated NSCLC for phase 1b study).
4. Patients with at least one measurable lesion as defined by Response Evaluation Criteria in Solid Tumors version 1.1 (RECIST 1.1) criteria.
5. At least 4 weeks must have lapsed from prior chemotherapy, immunotherapy or radiotherapy; at least 2 weeks or 5 half-lives must have lapsed from prior small-molecule target therapies (whichever was longer). All toxicities (except for alopecia and skin pigmentation) resolved (≤ grade 1 or baseline).
6. At least 4 weeks must have lapsed from prior major surgery (except for tumor biopsy) or severe trauma.
7. Eastern Cooperative Oncology Group (ECOG) performance status score of 0 or 1 at study entry.
8. Life expectancy ≥3 months.
9. Able to take the study drugs orally.
10. Laboratory tests within 7 days prior to study treatment:
    - Absolute neutrophil count ≥1.5×10^9^/L.
    - Platelet count ≥100×10^9^/L or lower limit of normal range.
    - Hemoglobin ≥9g/dL

14 days before the first dosing, hematological values maintained as above without treatments of G-CSF, platelet infusion, TPO, blood infusion, or supportive therapy of erythropoietin.

- - Creatinine ≤1.5×ULN or creatinine clearance >60 mL/min calculated by Cockroft-Gault formula.
  - AST/ALT ≤3×ULN; AST/ALT ≤5×ULN for patients with liver metastases.
  - Total bilirubin level ≤1.5×ULN; total bilirubin level ≤3×ULN for patients with Gilbert's syndrome.
  - Albumin ≥3g/dL.
  - International Normalized Ratio (INR) or plasma prothrombin time (PT) ≤1.5×ULN.
  - Creatine kinase (CK) ≤1.5×ULN.

1. Understand and voluntarily sign the ICF before the conduction of any study procedures.
2. Must be voluntary and able to complete study procedures and follow-up examinations.

Exclusion criteria

1. Known hypersensitivity to study drug ingredients or their analogues.
2. Had received specific MEK inhibitors and BRAF inhibitors.
3. Had received other study treatment within 4 weeks prior to current study treatment.
4. Had received other antitumor therapies (except for hormonal therapies) at the same time.
5. Patients with active central nervous system (CNS) lesion (which was imaging instability and symptomatic damage). Note: Patients received stereotactic brain radiotherapy or surgery had no brain disease progression for ≥1 month, and patients received surgery had no disease progression for ≥3 months could be enrolled.
6. Patients who were receiving and cannot discontinue prohibited intravenous or oral medications affecting CYP isozymes (strong inducers or strong inhibitors of CYP2C9 and CYP2C19) at least 1 week prior to the initiation of study treatment and during the study.
7. Bleeding symptoms of the National Cancer Institute Common Terminology Criteria for Adverse Events (NCI CTCAE) version 5·0 (NCI CTCAE v5.0) grade 3 within 4 weeks prior to the initiation of study treatment.
8. Inability to swallow capsules, refractory nausea and vomiting, malabsorption, extracorporeal bile diversion, or any significant small bowel resection that may interfere adequate absorption of study drug.
9. Patients had Bazett’s formula-corrected electrocardiography (ECG) QTcB ≥480 msec at screening, or a history of congenital long QT syndrome.
10. Any of the following situations occurred within 6 months prior to study treatment: myocardial infarction, severe/unstable angina, coronary/peripheral artery bypass grafting, symptomatic congestive heart failure, severe arrhythmia requiring medication, uncontrolled hypertension, cerebrovascular accident, or symptomatic pulmonary embolism.
11. Uncontrolled concomitant diseases or infectious diseases.
12. Prior or current retinal diseases, such as: central retinal vein occlusion (RVO), retinal artery occlusion (RAO), retinal vasculitis, retinal telangiectasia (Coats disease), retinal pigment epithelial detachment (RPED), etc.
13. Patients with human immunodeficiency virus (HIV) antibody positive, hepatitis C virus (HCV) antibody positive and HCV RNA positive, hepatitis B virus surface antigen (HBsAg) positive and HBV DNA positive (HBsAg positive required further test of HBV DNA, HBV DNA ≥200 IU/ml, or ≥10^3^ copies/ml).
14. Known history of chronic liver diseases.
15. History of allogeneic bone marrow or organ transplantation.
16. Patients with interstitial lung disease or interstitial pneumonitis, including patients with clinically significant radiation pneumonitis (ie, affecting activities of daily life or requiring intervention).
17. History of any other malignancy within 3 years (except for effectively controlled non-melanoma skin basal cell or squamous cell carcinoma, cervical carcinoma in situ, and other malignancies that were effectively controlled without treatment within the past 3 years).
18. Premenopausal women (postmenopausal women must have been menopausal for at least 12 months to be considered infertile) with a positive serum pregnancy test result, or patients of childbearing age who were expected to become pregnant, breastfeeding, or unwilling to use effective contraception (including female spouses at childbearing age of male patients) at the discretion of the investigator during the study and for at least 30 days after the last dose of study drug.
19. Any other severe, acute, or chronic clinical, psychiatric disorders, or laboratory abnormalities that might increase the risk of study participation and study medication or interfere with the interpretation of study results.

## Definition of secondary efficacy end points

ORR: proportion of patients with CR or PR

DoR: the time from the first documented CR or PR until the first documented PD or death from any cause),

DCR: proportion of patients with CR, PR, or SD

PFS: the time from the initiation of treatment to the occurrence of PD or death from any cause

CR, complete response; PR, partial response; SD, stable disease; PD, progressive disease.

## Measurement of PK

Blood samples were collected in EDTA pre-dose, and at 0.25, 0.5, 1, 2, 4, 8, and 12h after tunlametinib and vemurafenib administration on cycle 1 day 1 and cycle 2 day1. Plasma concentrations of tunlametinib and vemurafenib were analyzed using a validated ultra-performance liquid chromatography-tandem mass spectrometry method. All samples analyzed within established storage stability periods.

Supplementary Table S1. Demographic and Baseline Characteristics of All Patients with Solid Tumors by Dose Group

| Study Drug Dose Groups | 0.5+960 BID | 6+960  BID | 9+960  BID | 12+960  BID | 15+960  BID | 9+720  BID | 12+720  BID | Total |
| --- | --- | --- | --- | --- | --- | --- | --- | --- |
| HL-085 dose (mg, BID) | 0.5 | 6 | 9 | 12 | 15 | 9 | 12 | – |
| Vemurafenib dose (mg, BID) | 960 | 960 | 960 | 960 | 960 | 720 | 720 | – |
| No. of patients | 3 | 3 | 4 | 15 | 3 | 20 | 24 | 72 |
| Median age, years (range) | 63 (58-66) | 60 (46-70) | 60 (52-65) | 58 (41-68) | 47 (33-54) | 57 (37-81) | 54 (32-66) | 57 (32-81) |
| Sex, n (%) |  |  |  |  |  |  |  |  |
| Male | 0 | 3 (100.0) | 2 (50.0) | 9 (60.0) | 2 (66.7) | 12 (60.0) | 11 (45.8) | 39 (54.2) |
| Female | 3 (100.0) | 0 | 2 (50.0) | 6 (40.0) | 1 (33.3) | 8 (40.0) | 13 (54.2) | 33 (45.8) |
| ECOG PS, n (%) |  |  |  |  |  |  |  |  |
| 0 | 2 (66.7) | 3 (100.0) | 1 (25.0) | 8 (53.3) | 0 | 7 (35.0) | 11 (45.8) | 32 (44.4) |
| 1 | 1 (33.3) | 0 | 3 (75.0) | 7 (46.7) | 3 (100.0) | 13 (65.0) | 13 (54.2) | 40 (55.6) |
| Types of solid tumor, n (%) |  |  |  |  |  |  |  |  |
| NSCLC | 1 (33.3) | 2 (66.7) | 0 | 7 (46.7) | 0 | 17 (85.0) | 9 (37.5) | 36 (50.0) |
| CRC | 1 (33.3) | 1 (33.3) | 2 (50.0) | 5 (33.3) | 1 (33.3) | 1 (5.0) | 14 (58.3) | 25 (34.7) |
| Melanoma | 1 (33.3) | 0 | 2 (50.0) | 2 (13.3) | 1 (33.3) | 0 | 0 | 6 (8.3) |
| PTC | 0 | 0 | 0 | 1 (6.7) | 0 | 2 (10.0) | 1 (4.2) | 4 (5.6) |
| PDAC | 0 | 0 | 0 | 0 | 1 (33.3) | 0 | 0 | 1 (1.4) |
| BRAF V600 mutation, n (%) | 3 (100.0) | 3 (100.0) | 4 (100.0) | 15 (100.0) | 3 (100.0) | 20 (100.0) | 24 (100.0) | 72 (100.0) |
| Had received prior chemotherapy, n (%) | 2 (66.7) | 2 (66.7) | 4 (100.0) | 10 (66.7) | 3 (100.0) | 10 (50.0) | 17 (70.8) | 48 (66.7) |
| Had received prior target therapy, n (%) | 0 | 1 (33.3) | 1 (25.0) | 5 (33.3) | 1 (33.3) | 5 (25.0) | 11 (45.8) | 24 (33.3) |
| Had received prior ICI, n (%) | 1 (33.3) | 0 | 1 (25.0) | 0 | 0 | 0 | 0 | 2 (2.8) |
| Had received prior radiotherapy, n (%) | 0 | 2 (66.7) | 1 (25.0) | 1 (6.7) | 1 (33.3) | 3 (15.0) | 4 (16.7) | 12 (16.7) |

Abbreviation: CRC, colorectal cancer; ECOG PS, Eastern Cooperative Oncology Group performance status; NSCLC, non-small cell lung cancer; PDAC, pancreatic ductal adenocarcinoma; PTC, papillary thyroid carcinoma.

Supplementary Table S2. Demographic and Baseline Characteristics of NSCLC Patients by Dose Group

| Study Drug Dose Groups | 0.5+960  BID | 6+960  BID | 12+960  BID | 9+720  BID | 12+720  BID | Total |
| --- | --- | --- | --- | --- | --- | --- |
| HL-085 dose (mg, BID) | 0.5 | 6 | 12 | 9 | 12 | – |
| Vemurafenib dose (mg, BID) | 960 | 960 | 960 | 720 | 720 | – |
| No. of patients | 1 | 2 | 7 | 17 | 9 | 36 |
| Median age, years (range) | 63 (63-63) | 65 (60-70) | 60 (53-67) | 59 (37-81) | 60 (49-66) | 60 (37-81) |
| Sex, n (%) |  |  |  |  |  |  |
| Male | 0 | 2 (100.0) | 5 (71.4) | 10 (58.8) | 2 (22.2) | 19 (52.8) |
| Female | 1 (100.0) | 0 | 2 (28.6) | 7 (41.2) | 7 (77.8) | 17 (47.2) |
| ECOG PS, n (%) |  |  |  |  |  |  |
| 0 | 1 (100.0) | 2 (100.0) | 3 (42.9) | 5 (29.4) | 5 (55.6) | 16 (44.4) |
| 1 | 0 | 0 | 4 (57.1) | 12 (70.6) | 4 (44.4) | 20 (55.6) |
| BRAF V600 mutation, n (%) | 1 (100.0) | 2 (100.0) | 7 (100.0) | 17 (100.0) | 9 (100.0) | 36 (100.0) |
| Had received prior chemotherapy, n (%) | 0 | 1 (50.0) | 3 (42.9) | 9 (52.9) | 4 (44.4) | 17 (47.2) |
| Had received prior target therapy, n (%) | 0 | 0 | 2 (28.6) | 5 (29.4) | 1 (11.1) | 8 (22.2) |
| Had received prior ICI, n (%) | 0 | 0 | 0 | 0 | 0 | 0 |
| Had received prior radiotherapy, n (%) | 0 | 1 (50.0) | 0 | 2 (11.8) | 3 (33.3) | 6 (16.7) |
| Abbreviation: ECOG PS, Eastern Cooperative Oncology Group performance status; ICI, immune checkpoint inhibitor. | | | | | | |

Supplementary Table S3. AE Summary of All, NSCLC, and CRC Patients

|  |  |  |  |  |  |  |  | All patients | NSCLC patients | CRC patients |
| --- | --- | --- | --- | --- | --- | --- | --- | --- | --- | --- |
| AEs, n (%) | 0.5+960 | 6+960 | 9+960 | 12+960 | 15+960 | 9+720 | 12+720 | Total | Total | Total |
| HL-085 dose  (mg, BID) | 0.5 | 6 | 9 | 12 | 15 | 9 | 12 | – | – | – |
| Vemurafenib dose (mg, BID) | 960 | 960 | 960 | 960 | 960 | 720 | 720 | – | – | – |
| No. of patients | 3 | 3 | 4 | 15 | 3 | 20 | 24 | 72 | 36 | 25 |
| Any Grade | 3 (100.0) | 3 (100.0) | 4 (100.0) | 15 (100.0) | 3 (100.0) | 20 (100.0) | 24 (100.0) | 72 (100.0) | 36 (100.0) | 25 (100.0) |
| ≥ Grade 3 TEAEs | 2 (66.7) | 2 (66.7) | 4 (100.0) | 9 (60.0) | 2 (66.7) | 12 (60.0) | 17 (70.8) | 48 (66.7) | 21 (58.3) | 18 (72.0) |
| Serious TEAEs | 1 (33.3) | 0 | 3 (75.0) | 6 (40.0) | 1 (33.3) | 8 (40.0) | 15 (62.5) | 34 (47.2) | 17 (47.2) | 13 (52.0) |
| TEAE leading to drug discontinuation | 0 | 0 | 1 (25.0) | 6 (40.0) | 2 (66.7) | 2 (10.0) | 0 | 11 (15.3) | 4 (11.1) | 1 (4.0) |
| TEAE leading to drug interruption | 1 (33.3) | 1 (33.3) | 4 (100.0) | 13 (86.7) | 2 (66.7) | 15 (75.0) | 22 (91.7) | 58 (80.6) | 26 (72.2) | 24 (96.0) |
| TEAE leading to death | 0 | 0 | 1 (25.0) | 0 | 0 | 2 (10.0) | 2 (8.3) | 5 (6.9) | 4 (11.1) | 0 |
| Any Grade of TRAEs | 3 (100.0) | 3 (100.0) | 4 (100.0) | 15 (100.0) | 3 (100.0) | 20 (100.0) | 24 (100.0) | 72 (100.0) | 36 (100.0) | 25 (100.0) |
| ≥ Grade 3 TRAEs | 2 (66.7) | 2 (66.7) | 4 (100.0) | 9 (60.0) | 2 (66.7) | 9 (45.0) | 15 (62.5) | 43 (59.7) | 16 (44.4) | 18 (72.0) |
| Serious TRAEs | 1 (33.3) | 0 | 2 (50.0) | 5 (33.3) | 1 (33.3) | 5 (25.0) | 9 (37.5) | 23 (31.9) | 10 (27.8) | 10 (40.0) |
| TRAE leading to drug discontinuation | 0 | 0 | 1 (25.0) | 4 (26.7) | 2 (66.7) | 1 (5.0) | 0 | 8 (11.1) | 1 (2.8) | 1 (4.0) |
| TRAE leading to drug interruption | 1 (33.3) | 1 (33.3) | 4 (100.0) | 13 (86.7) | 2 (66.7) | 15 (75.0) | 21 (87.5) | 57 (79.2) | 25 (69.4) | 24 (96.0) |
| TRAE leading to death | 0 | 0 | 0 | 0 | 0 | 0 | 1 (4.2) | 1 (1.4) | 1 (2.8) | 0 |
| Abbreviation: AE, adverse event; CRC, colorectal cancer; NSCLC, non-small cell lung cancer; TEAEs, Treatment-Emergent Adverse Events; TRAEs, Treatment-Related Adverse Events. | | | | | | | | | | |

Supplementary Table S4. Antitumor activities of Tunlametinib/Vemurafenib in CRC and PTC Patients with Evaluable Disease

| Groups | 0.5+960  BID | 6+960  BID | 9+960  BID | 12+960  BID | 15+960  BID | 9+720  BID | 12+720  BID | Total |
| --- | --- | --- | --- | --- | --- | --- | --- | --- |
| HL-085 dose (mg, BID) | 0.5 | 6 | 9 | 12 | 15 | 9 | 12 | – |
| Vemurafenib dose  (mg, BID) | 960 | 960 | 960 | 960 | 960 | 720 | 720 | – |
| CRC patients |  |  |  |  |  |  |  |  |
| No. of patients | 1 | 1 | 1 | 5 | 1 | 1 | 14 | 24 |
| ORR, n (%) | 0 | 1 (100.0) | 0 | 1 (20.0) | 0 | 0 | 4 (28.6) | 6 (25.0) |
| PR | 0 | 1 (100.0) | 0 | 1 (20.0) | 0 | 0 | 4 (28.6) | 6 (25.0) |
| SD | 1 (100.0) | 0 | 1 (100.0) | 3 (60.0) | 1 (100.0) | 1 (100.0) | 8 (57.1) | 15 (62.5) |
| PD | 0 | 0 | 0 | 1 (20.0) | 0 | 0 | 1 (7.1) | 2 (8.3) |
| NE | 0 | 0 | 0 | 0 | 0 | 0 | 1 (7.1) | 1 (4.2) |
| DCR, n (%) | 1 (100.0) | 1 (100.0) | 1 (100.0) | 4 (80.0) | 1 (100.0) | 1 (100.0) | 12 (85.7) | 21 (87.5) |
| Median PFS, months (95% CI) | – | – |  | – |  | – | – | 6.2 (4.8-7.6) |
| Median DoR, months (95% CI) | – | – |  | – |  | – | – | 5.5 (2.9-NE) |
| PTC patients |  |  |  |  |  |  |  |  |
| No. of patients | 0 | 0 | 0 | 1 | 0 | 2 | 1 | 4 |
| ORR, n (%) | 0 | 0 | 0 | 1 (100.0) | 0 | 1 (50.0) | 0 | 2 (50.0) |
| DCR, n (%) | 0 | 0 | 0 | 1 (100.0) | 0 | 1 (100.0) | 1 (100.0) | 1 (100.0) |
| Abbreviation: CRC, colorectal cancer; CR, complete response; PR, partial response; SD, stable disease; PD, progressive disease; PTC, papillary thyroid carcinomas; NE, not evaluable; NR, not reached; ORR, objective response rate; CI, confidence interval; DCR, disease control rate. | | | | | | | | |

Supplementary Table S5. PK parameters of Tunlametinib (0.5mg-15mg)

|  | Parameters | Units | Tunlametinib  0.5mg BID + vemurafenib 960mg BID | Tunlametinib  6mg BID + vemurafenib 960mg BID | Tunlametinib 9mg BID | | | Tunlametinib 12mg BID | | | Tunlametinib 15mg BID + vemurafenib 960mg BID |
| --- | --- | --- | --- | --- | --- | --- | --- | --- | --- | --- | --- |
|  |  |  |  |  | Tunlametinib 9mg BID | Tunlametinib 9mg BID + vemurafenib 720mg BID | Tunlametinib 9mg BID + vemurafenib 960mg BID | Tunlametinib 12mg BID | Tunlametinib 12mg BID + vemurafenib 720mg BID | Tunlametinib 12mg BID +vemurafenib 960mg BID |  |
| Single dose | AUC_last_ | hng/mL | 7.45 (2.75) | 90.13 (29.83) | 160.58 (62.24) | 154.25 (47.15) | 173.23 (93.25) | 232.73 (84.64) | 253.53 (72.26) | 203.6 (94.29) | 301.83 (44.29) |
|  | AUC_tau_ | hng/mL | 6.68 (3.39) | 90.80 (29.95) | 161.58 (62.52) | 155.07 (47.49) | 174.59 (93.44) | 230.82 (86.45) | 250.55 (72.00) | 194.95 (101.87) | 304.26 (44.4) |
|  | C_max_ | ng/mL | 2.16 (0.99) | 53.53 (22.78) | 82.05 (58.08) | 88.85 (62.61) | 68.45 (53.42) | 108.78 (70.48) | 131.54 (71.11) | 76.92 (57.7) | 99.03 (35.05) |
|  | T_max_ | h | 1.00 (1.00-3.93) | 0.50 (0.28-0.50) | 0.97 (0.28-2.07) | 0.73 (0.28-2.07) | 0.99 (0.50-1.05) | 0.50 (0.28-4.08) | 0.50 (0.28-4.08) | 0.52 (0.28-4.08) | 1.00 (0.92-2.05) |
| Multiple dose | AUC_last_ | hng/mL | 22.45 (2.91) | 211.55 (14.38) | 351.74 (120.84) | 309.65 (107.00) | 399.84 (125.13) | 384.23 (96.83) | 414.08 (90.05) | 388.71 (39.47) | 470.69 (24.54) |
|  | AUC_tau_ | hng/mL | 22.91 (3.12) | 230.62 (NC) | 351.62 (125.99) | 297.87 (108) | 405.36 (126.44) | 389.49 (101.07) | 416.88 (91.10) | 409.75 (35.10) | 473.10 (26.84) |
|  | C_avg_ | ng/mL | 1.91 (0.26) | 19.22 (NC) | 29.3 (10.5) | 24.82 (9.00) | 33.78 (10.54) | 32.46 (8.42) | 34.74 (7.59) | 34.15 (2.92) | 39.42 (2.24) |
|  | C_max_ | ng/mL | 5.99 (1.54) | 37.30 (15.50) | 97.13 (75.03) | 106.50 (97.25) | 86.43 (43.03) | 109.82 (36.89) | 126.62 (33.89) | 83.80 (23.53) | 135.33 (26.50) |
|  | Fluctuation% | % | 267.46 (108.39) | 238.31 (NC) | 306.79 (201.04) | 379.40 (225.69) | 234.18 (155.98) | 328.83 (79.83) | 343.03 (81.17) | 249.48 (85.32) | 313.39 (46.19) |
|  | R_AUClast_ | - | 3.52 (2.06) | 2.82 (0.74) | 2.15 (0.61) | 1.97 (0.63) | 2.51 (0.45) | 1.89 (0.55) | 1.71 (0.32) | 2.63 (0.23) | 1.59 (0.33) |
|  | R_AUCtau_ | - | 4.30 (2.54) | NC (NC) | 2.08 (0.60) | 1.82 (0.56) | 2.52 (0.44) | 1.81 (0.58) | 1.72 (0.37) | 2.89 (NC) | 1.59 (0.33) |
|  | R_Cmax_ | - | 3.01 (1.11) | 0.71 (0.06) | 1.39 (0.88) | 1.03 (0.63) | 2.12 (0.97) | 1.49 (0.72) | 1.34 (0.73) | 1.77 (0.84) | 1.47 (0.52) |
|  | T_max_ | h | 0.50  (0.50-0.53) | 3.98  (1.03-4.00) | 1.02  (0.48-3.98) | 1.00  (0.48-3.98) | 1.92  (0.95-2.00) | 1.48  (0.48-4.00) | 1.00  (0.53-2.00) | 2.03  (2.00-4.00) | 2.02  (0.95-2.05) |

*：Represents the dose of tunlametinib and vemurafeinib, respectively. NC：Not calculated. All the PK parameters shown as Mean±SD, except T_max_ shown as Median (range).

Supplementary Table S6. PK parameters of Vemurafenib (720mg-960mg)

|  | Parameters | Units | Vemurafenib 720mg BID | | | Vemurafenib 960mg BID | | |
| --- | --- | --- | --- | --- | --- | --- | --- | --- |
|  |  |  | Vemurafenib 720mg BID Total  (Tunlametinib 9mg BID + vemurafenib 720mg BID, Tunlametinib 12mg BID + vemurafenib 720mg BID) | Tunlametinib 9mg BID + vemurafenib 720mg BID | Tunlametinib 12mg BID + vemurafenib 720mg BID | 960mg Total  (Tunlametinib 0.5mg BID + vemurafenib 960mg BID, Tunlametinib 6mg BID + vemurafenib 960mg BID, Tunlametinib 9mg BID + vemurafenib 960mg BID, Tunlametinib 12mg BID + vemurafenib 960mg BID, Tunlametinib 15mg BID + vemurafenib 960mg BID) | Tunlametinib 9mg BID + vemurafenib 960mg BID | Tunlametinib 12mg BID + vemurafenib 960mg BID |
| Single dose | AUC__%Extrap_ | % | 60.37 (6.66) | 56.66 (6.57) | 61.30 (6.78) | 61.29 (11.94) | 57.98 (NC) | 64.58 (NC) |
|  | AUC_last_ | h*ng/mL | 39348.91 (20252.46) | 45824.27 (21581.75) | 37190.46 (19860.01) | 50526.59 (30382.19) | 65323.11 (67573.89) | 47073.78 (19278.3) |
|  | AUC_tau_ | h*ng/mL | 41007.33 (15133.07) | 55194.80 (2015.80) | 38169.84 (15025.77) | 61199.87 (29324.34) | 117962.86 (NC) | 37690.87 (NC) |
|  | C_max_ | ng/mL | 4888.21 (2431.37) | 5711.43 (2385.78) | 4613.81 (2440.65) | 6215.39 (3482.68) | 7363.25 (7237.57) | 5714.67 (2253.39) |
|  | T_max_ | h | 4.00 (1.97-7.98) | 4.00 (1.97-4.03) | 4.00 (1.97-7.98) | 4.01 (1.98-11.57) | 3.96 (2.05-7.67) | 4.07 (2.08-11.57) |
| Multiple dose | AUC_last_ | h*ng/mL | 372099.65 (181007.44) | 375638.01 (164057.99) | 378548.97 (212322.47) | 503132.66 (192460.83) | 529672.28 (214509.71) | 455866.25 (151525.43) |
|  | AUC_tau_ | h*ng/mL | 391827.15 (191505.76) | 357513.69 (13113.86) | 408983.88 (244723.02) | 542735.68 (213630.85) | 565364.82 (NC) | 339897.94 (NC) |
|  | C_avg_ | ng/mL | 32652.26 (15958.81) | 29792.81 (1092.82) | 34081.99 (20393.59) | 45227.97 (17802.57) | 47113.74 (NC) | 28324.83 (NC) |
|  | C_max_ | ng/mL | 36555.00 (17439.27) | 37387.50 (14688.72) | 36843.33 (21118.48) | 48343.75 (19381.71) | 49171.43 (19088.98) | 42766.67 (11578.57) |
|  | Fluctuation% | % | 36.71 (12.64) | 37.17 (4.45) | 36.48 (16.11) | 29.65 (16.23) | 19.95 (NC) | 25.42 (NC) |
|  | R_AUClast_ | - | 11.10 (4.84) | 11.76 (5.15) | 10.61 (4.88) | 23.92 (39.19) | 36.26 (58.5) | 10.75 (2.55) |
|  | R_AUCtau_ | - | 13.29 (4.87) | NC (NC) | 13.29 (4.87) | 14.07 (NC) | NC (NC) | NC (NC) |
|  | R_Cmax_ | - | 8.70 (3.77) | 8.92 (3.76) | 8.54 (4.03) | 13.22 (11.54) | 16.01 (16.13) | 8.23 (1.79) |
|  | T_max_ | h | 2.07 (0.00-11.70) | 3.92 (0.00-8.00) | 1.93 (0.00-11.70) | 3.99 (0.00-8.32) | 4.07 (0.00-8.32) | 3.95 (0.00-3.98) |

*：Represents the dose of tunlametinib and vemurafeinib, respectively. NC：Not calculated. All the PK parameters shown as Mean±SD, except T_max_ shown as Median (range).

**Supplementary Fig S1.** Tumor responses in patient with evaluable disease. Best percentage change from baseline in total sum of target lesion diameters. (A) tunlametinib 9 mg BID plus vemurafenib 720 mg BID dose group in NSCLC patients, (B) CRC patients, (C) PTC patients, (D) Melanoma and PDAC patients. All dose groups were given study drug treatment twice daily (BID). BID, Bis In Die; CR, complete response; CRC, colorectal cancer; NSCLC, non–small-cell lung cancer; NE, not evaluable; PDAC, pancreatic ductal adenocarcinoma; PTC, papillary thyroid carcinoma; PD, progressive disease; PR, partial response; SD, stable disease. Note: Among them, 7 patients were presented in Figure S1D, of which 01007 was PDAC and all others were Melanoma.


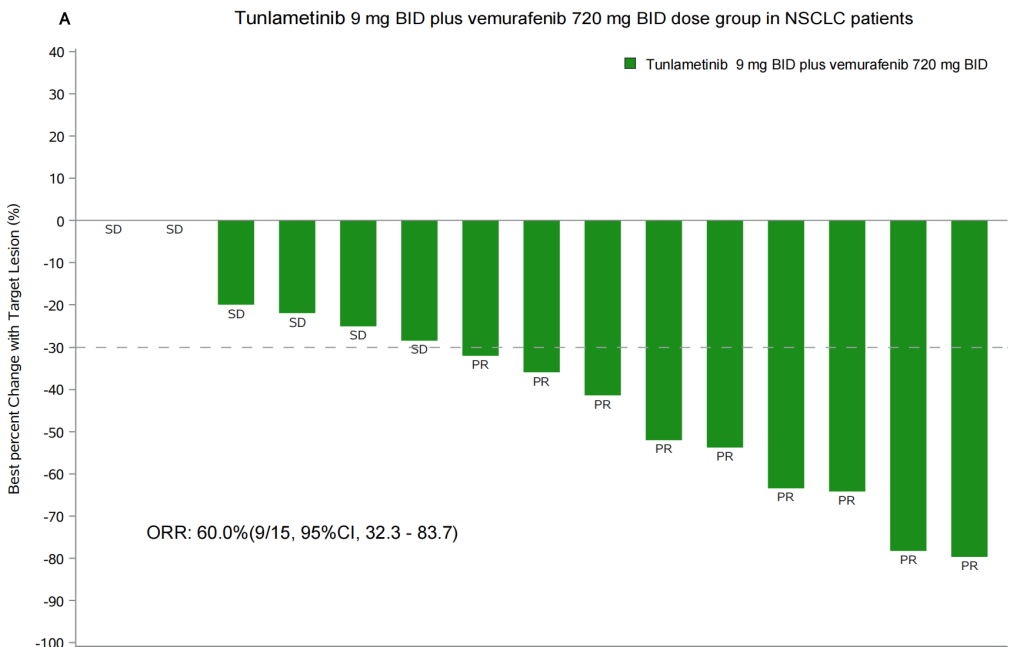

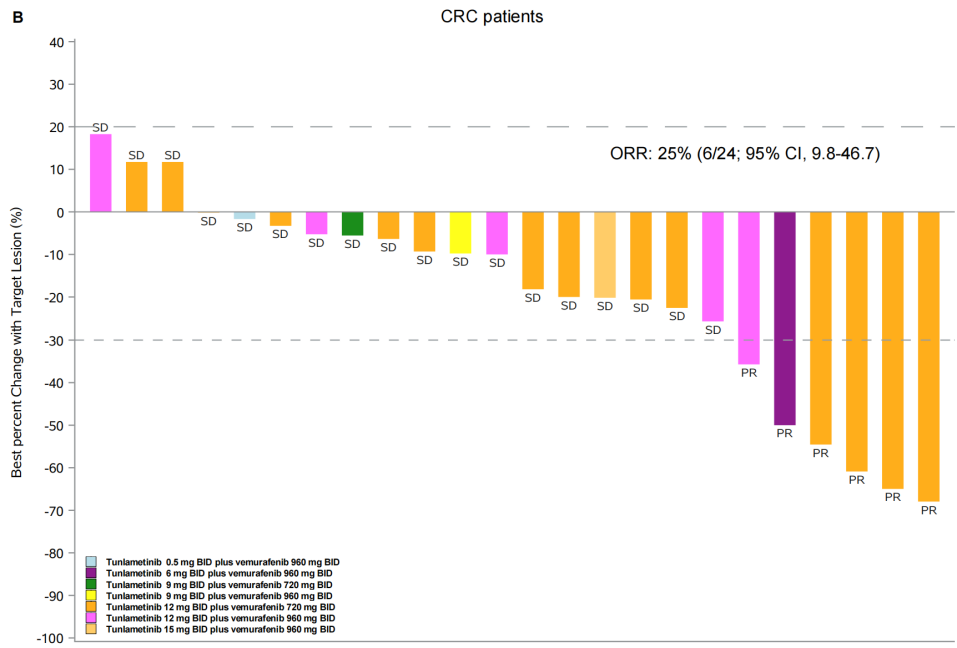


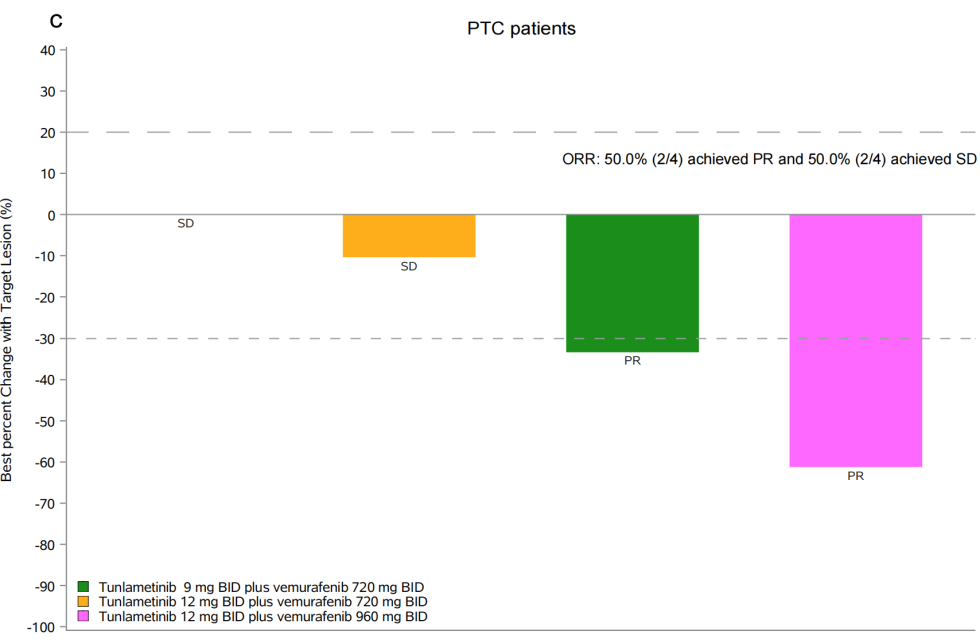

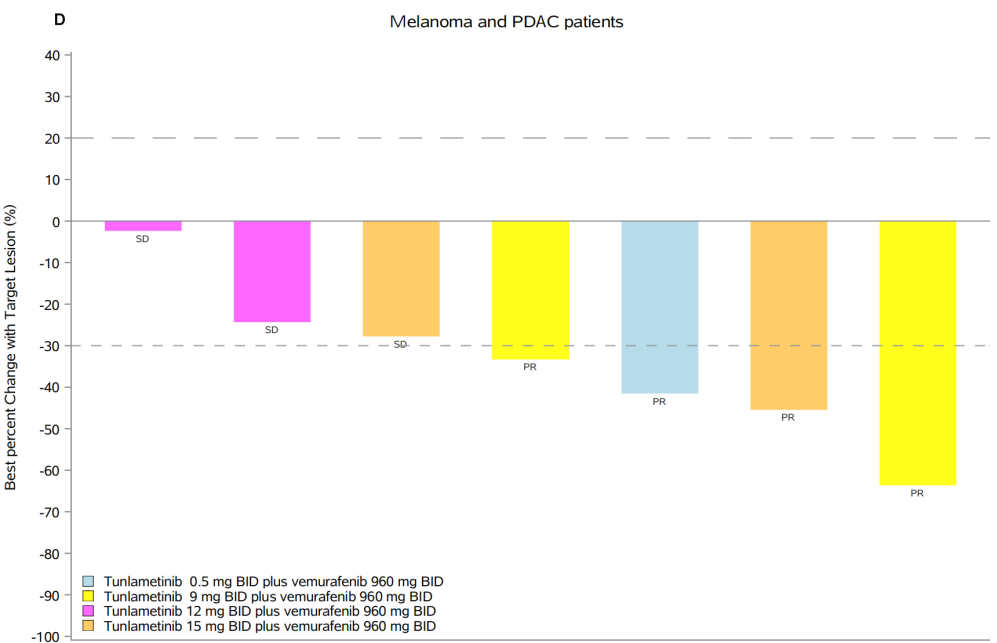


Supplementary Fig S2. Treatment duration of patient with evaluable disease. (A) tunlametinib 9 mg BID plus vemurafenib 720 mg BID dose group in NSCLC patients, (B) CRC patients, (C) PTC patients and (D) Melanoma and PDAC patients. All dose groups were given study drug treatment twice daily (BID). BID, Bis In Die; CR, complete response; CRC, colorectal cancer; NSCLC, non–small-cell lung cancer; NE, not evaluable; PDAC, pancreatic ductal adenocarcinoma; PTC, papillary thyroid carcinoma; PD, progressive disease; PR, partial response; SD, stable disease. Note: Among them, 7 patients were presented in Figure S2D, of which 01007 was PDAC and all others were Melanoma.


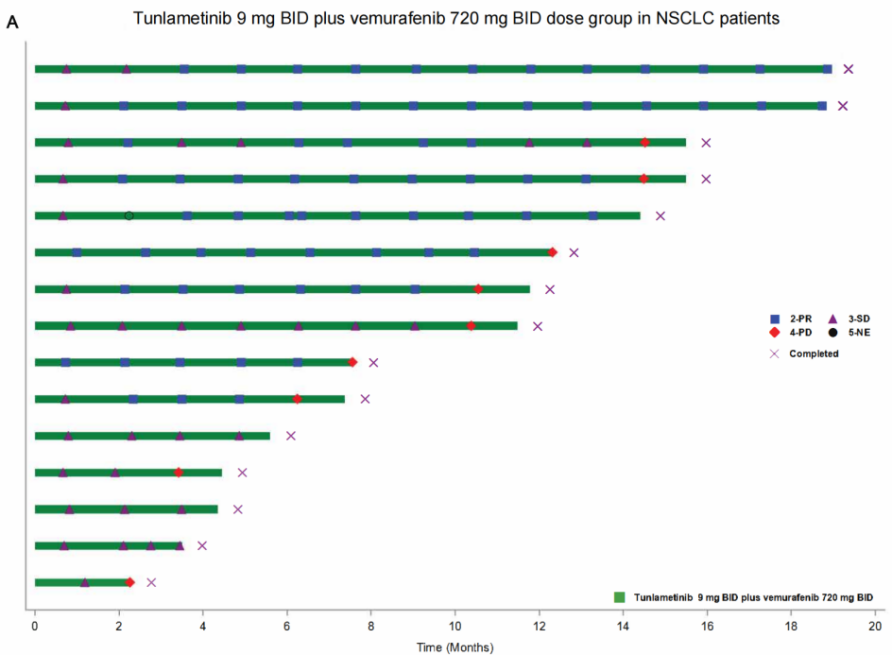

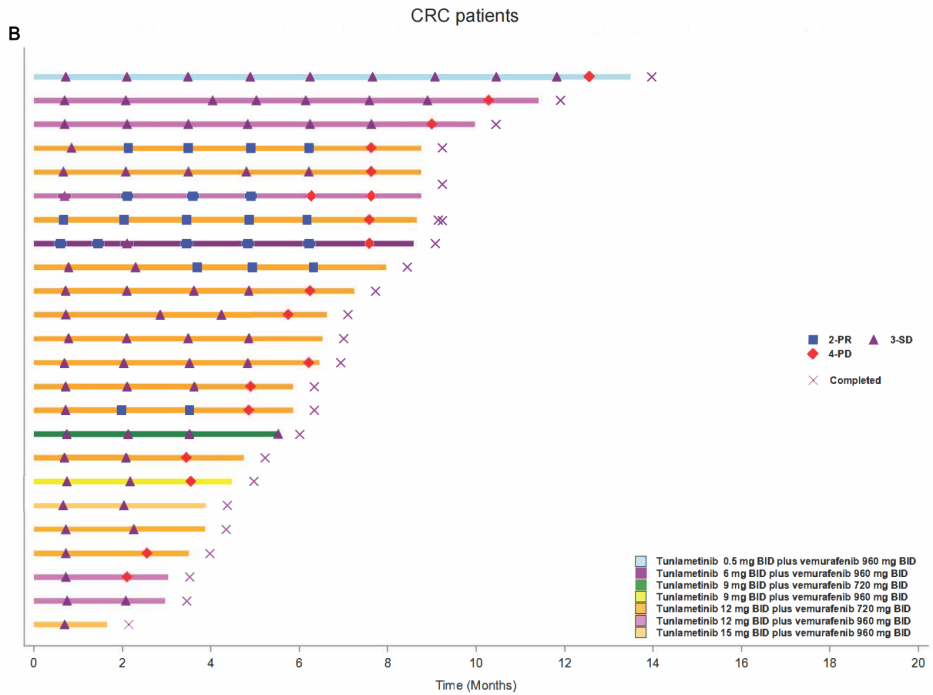


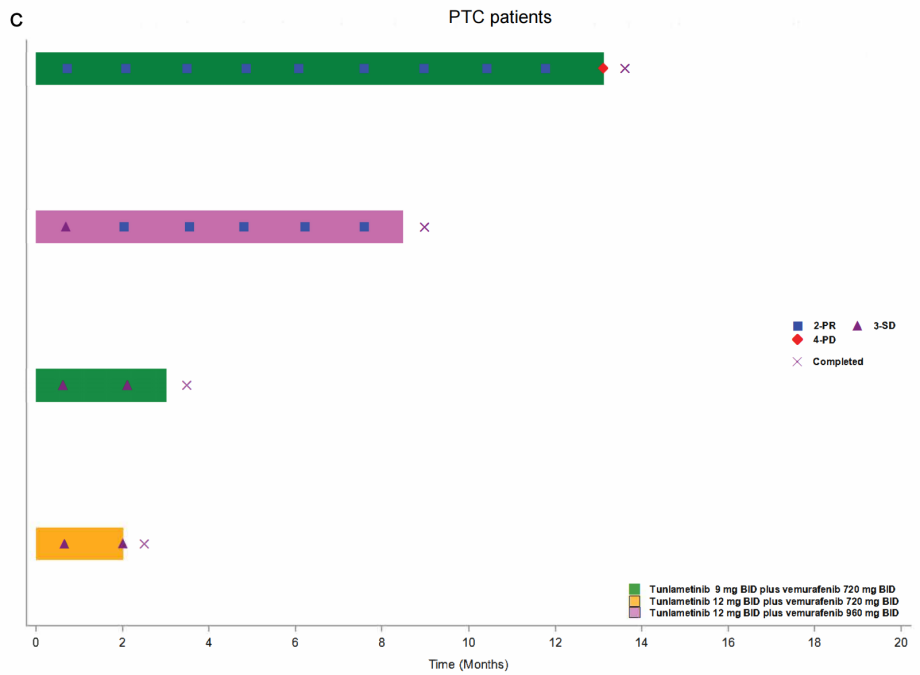

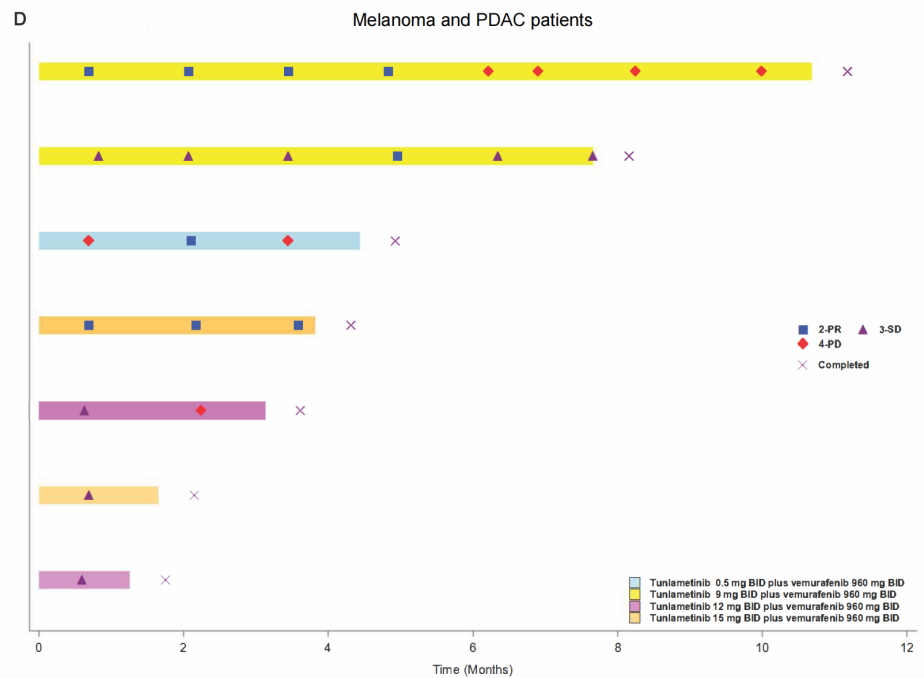


**Supplementary Fig S3.** Kaplan-Meier curves. (A) Progression-free survival in CRC patients; (B) Duration of response in CRC patients. CRC, colorectal cancer; PFS, progression-free survival; DoR, duration of response.


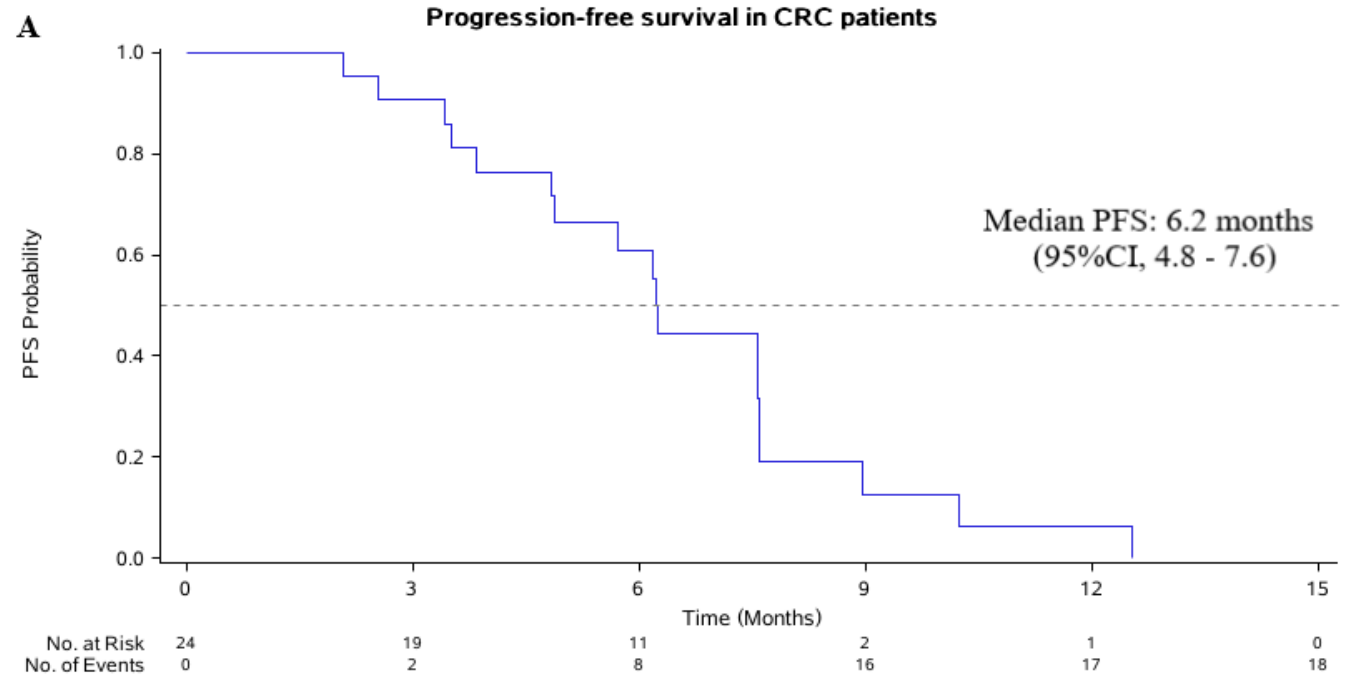


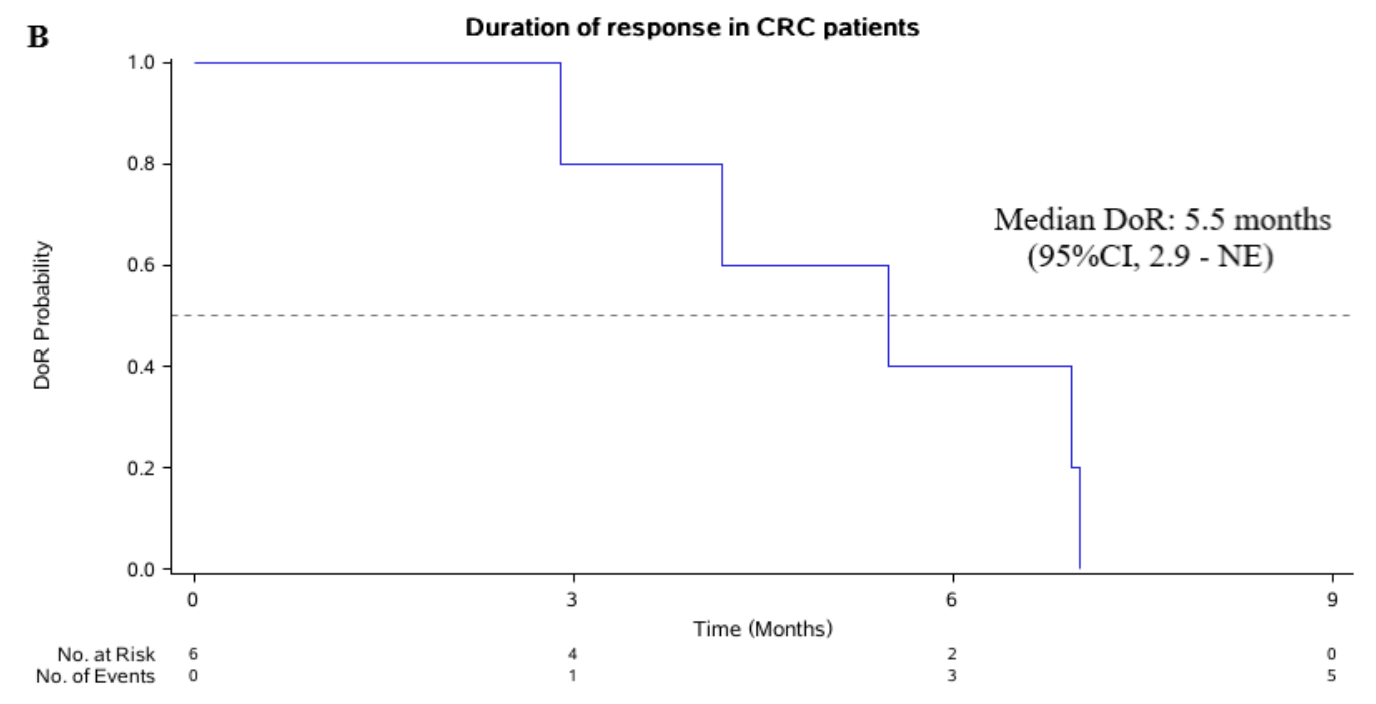


**Supplementary Fig S4.** Responses of all patients with evaluable disease. (A) Waterfall plot of best percentage change from baseline in total sum of target lesion diameters. (B) Swimmer plot of study drug treatment duration. CR, complete response; PR, partial response; SD, stable disease; PD, progressive disease.


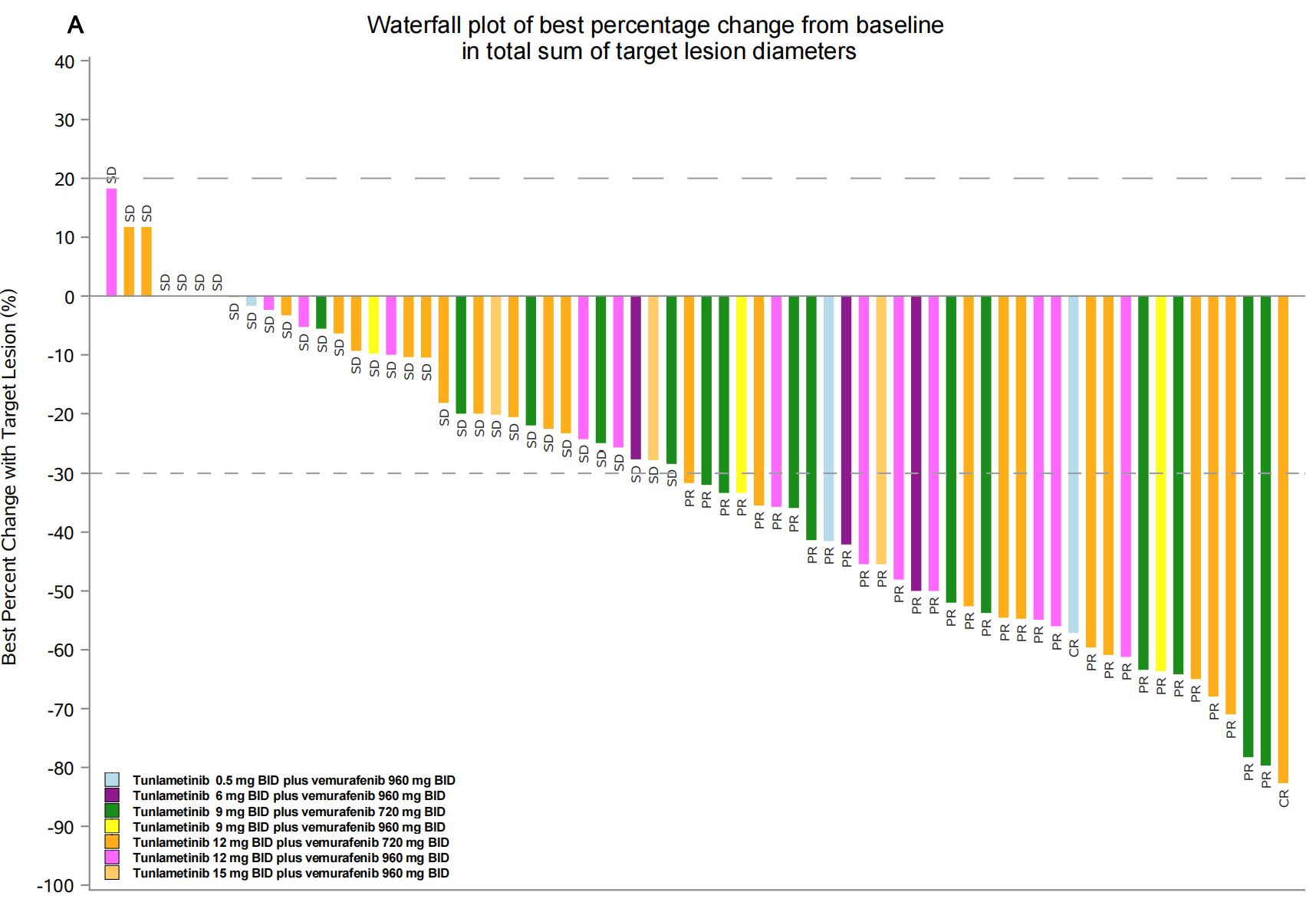


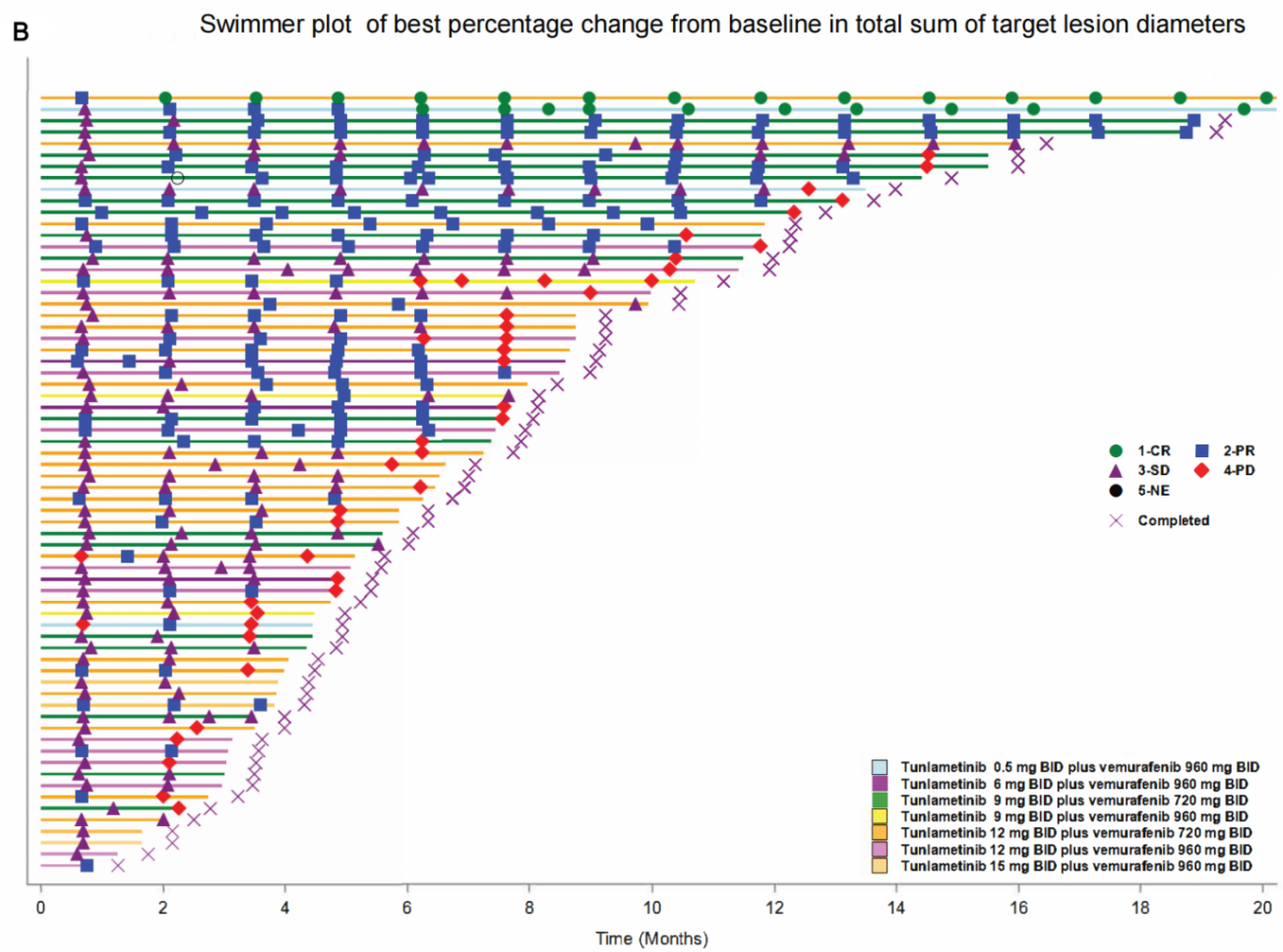

Supplement: Supplementary file 1 — Supplementary Material 1 [file 40164_2024_528_MOESM1_ESM.docx]
